# Associations between *XRCC3* Thr241Met polymorphisms and breast cancer risk: systematic-review and meta-analysis of 55 case-control studies

**DOI:** 10.1186/s12881-019-0809-8

**Published:** 2019-05-10

**Authors:** Sepideh Dashti, Zahra Taherian-Esfahani, Abbasali Keshtkar, Soudeh Ghafouri-Fard

**Affiliations:** 1 Department of Medical Genetics, Shahid Beheshti University of Medical Sciences, Tehran, Iran; 2 Department of Health Sciences Education Development, School of Public Health, Tehran University of Medical Sciences, Tehran, Iran

**Keywords:** Genes, Neoplasm, Single nucleotide polymorphism, Breast Cancer

## Abstract

**Background:**

The *X-ray repair cross-complementing group 3* (*XRCC3*) is an efficient component of homologous recombination and is required for the preservation of chromosomal integrity in mammalian cells. The association between Thr241Met single-nucleotide polymorphism (SNP) in this gene and susceptibility to breast cancer has been assessed in several studies. Yet, reports are controversial. The present meta-analysis has been designed to identify whether this SNP is associated with susceptibility to breast cancer.

**Methods:**

We performed a systematic review and meta-analysis for retrieving the case-control studies on the associations between T241 M SNP and the risk of breast cancer. Crude odds ratios (ORs) and 95% confidence intervals (CIs) were calculated to verify the association in dominant, recessive, and homozygote inheritance models.

**Results:**

We included 55 studies containing 30,966 sporadic breast cancer cases, 1174 familial breast cancer cases and 32,890 controls in the meta-analysis. In crude analyses, no association was detected between the mentioned SNP and breast cancer risk in recessive, homozygote or dominant models. However, ethnic based analysis showed that in sporadic breast cancer, the SNP was associated with breast cancer risk in Arab populations in homozygous (OR (95% CI) = 3.649 (2.029–6.563), *p* = 0.0001) and recessive models (OR (95% CI) = 4.092 (1.806–9.271), *p* = 0.001). The association was significant in Asian population in dominant model (OR (95% CI) = 1.296, *p* = 0.029). However, the associations was significant in familial breast cancer in mixed ethnic-based subgroup in homozygote and recessive models (OR (95% CI) = 0.451 (0.309–0.659), *p* = 0.0001, OR (95% CI) = 0.462 (0.298–0.716), *p* = 0.001 respectively).

**Conclusions:**

Taken together, our results in a large sample of both sporadic and familial cases of breast cancer showed insignificant role of Thr241Met in the pathogenesis of this type of malignancy. Such results were more conclusive in sporadic cases. In familial cases, future studies are needed to verify our results.

**Electronic supplementary material:**

The online version of this article (10.1186/s12881-019-0809-8) contains supplementary material, which is available to authorized users.

## Background

Breast cancer ranks first among all women’s cancers regarding its incidence and rank second among them regarding its cancer-related mortality rate [1]. Several genetic and environmental factors have been associated with breast cancer risk. Among the most relevant factors is the ability to repair DNA double strand break (DSB). The homologous recombination (HR) and the non-homologous end-joining (NHEJ) pathways have been developed in eukaryotic cells for repair of such defects [2]. Numerous single nucleotide polymorphisms (SNPs) within genes coding the NHEJ pathway have been associated with breast cancer risk [3]. More importantly, the mostly recognized breast cancer susceptibility genes *BRCA1* and *BRCA2* participate in the process of HR. Deficiencies in HR have been detected both in *BRCA1*/2 germline mutation–associated and remarkable fraction BRCA1/2 wild-type breast cancer patients [4]. The *X-ray repair cross-complementing group 3* (*XRCC3*) is an efficient component of HR and is required for the preservation of chromosomal integrity in mammalian cells [5]. Consequently, it has been regarded as a supposed candidate gene for breast cancer susceptibility. However, the data regarding its participation in breast cancer risk are inconsistent. Hang et al. conducted a meta-analysis of 48 case-control studies (including 14 studies in breast cancer) and reported that *XRCC3* Thr241Met significantly increased risk of breast cancer. However, they suggested that a single larger study should be performed to assess tissue-specific cancer risk in different ethnicities [6]. Garcı’a-Closas et al. meta-analyzed the studies in Caucasian populations (10,979 cases and 10,423 controls) and reported a weak association between homozygous variants for *XRCC3* Thr241Met and risk of breast cancer. They concluded that this variant is implausible to have a considerable role in breast cancer risk. However, they suggested studies with larger sample sizes to assess probable underlying gene–gene interactions or associations in ethnic-based subgroups [7]. Lee et al. in their meta-analysis of 12 studies demonstrated that Thr/Met and Met/Met weakly elevated the risk of breast cancer compared to Thr/Thr genotype [8]. Economopoulos et al. conducted a meta-analysis on 20 case–control studies in non-Chinese individuals and three case–control studies on Chinese individuals and reported association between T allele of this polymorphism (corresponding to Met) and breast cancer risk in recessive model. However, the association was only detected in non-Chinese population [9]. He et al. reported the mentioned association in recessive and additive models, but suggested conduction of a study with the larger sample size to assess gene-environment interaction [10]. In another study, He et al. have conducted a meta-analysis of 157 case-control studies including 34 studies in breast cancer (22,917 cases and 24,313 controls) and suggested the *XRCC3* Thr241Met as a susceptibility locus for breast cancer, especially in Caucasians [11]. Mao et al. demonstrated a significantly higher risk of breast cancer in heterozygote model but not in other models. Such association was significant in Asians. Based on the reported weak association, they suggested conduction of a study with larger sample size [12]. Finally, using 23 case-control studies, Chai et al. reported association between the mentioned polymorphism and breast cancer risk, especially in Asian populations and in patients without family history of breast cancer [13].

Therefore, according to inconclusive results of the previous meta-analyses and lack of systematic review in this regard, we conducted a systematic review and meta-analysis to assess the association between the Thr241Met SNP (rs861539) within *XRCC3* and breast cancer risk in diverse inheritance models.

## Methods

### Registration

We conducted the present systematic review protocol according to the preferred reporting items for systematic review and meta-analysis protocols (PRISMA-P) [14]. We also registered the study protocol on the international prospective register of systematic review (PROSPERO) network. The registration number was CRD42018104217.

### Information source and searching strategy

We searched PubMed, Scopus, EMBASE, Web of Science and ProQuest databases, the key journals (Breast Cancer Research and Treatment, Cancer Research), conferences/ congress research papers (as Grey literature) and the reference list of the included primary studies until March 2018 T(1990/01/01:2018/03/31) using the following syntaxes: “x-ray repair cross-complementing group 3” or “XRCC3”and“polymorphisms” or “single nucleotide polymorphism” and “breast tumor” or “breast cancer” and “rs861539” or “c.722C > T” or “p.Thr241Met” or “T241 M” (see Additional file 1). The complete search syntaxes were developed based on MeSH database and Emtree. The syntaxes for each database are shown in supplementary file. We did not implement any language restriction.

### Eligibility criteria and selection process

We included: i) all observational studies such as cross-sectional, case-control and cohort studies ii) studies that assessed associations between Thr241Met within *XRCC3* and breast cancer risk. iii) Studies with available genotype frequencies in both case and control groups. We excluded books, reviews, editorial, letters and articles which did not intend to assess the association between *XRCC3* Thr241Met SNP and breast cancer risk and those without control group data. Our participants are post- or pre-menopause women with breast cancer which is pathologically confirmed. Studies with male breast cancer cases were excluded. Our exposure is rs861539 (T241 M) that was evaluated with various genotyping methods such as PCR-RFLP, Taq-Man, Sequencing and etc. We performed search in the different mentioned sources and exported the search outputs into the End-Note software. The duplicated primary studies were deleted (only one version of the duplicated documents was kept). The screening phase (selecting included/ probable included versus excluded primary studies using the title or/ and the abstract) were performed. The selection or verification process (selecting included versus excluded primary studies) were performed based on the eligibility criteria. All steps for preparing this systematic review such as searching, screening based on titles of papers and abstracts, selection according to examination of full text of articles, risk of bias assessment and data extraction were done independently by two authors (SD and ZTE). Any disagreement regarding the inclusion/exclusion criteria and data extraction were resolved by consensus of the reviewers.

### Quality assessment and data extraction

Methodological quality assessment (risk of bias assessment) was based on the Newcastle–Ottawa Scale (NOS). Checklist of each study was filled with two reviewers independently. Any disagreements (between two reviewers) were resolved by the discussion or consensus otherwise opinion of third expert reviewer. For assessing total quality status in primary study we used sum score of quality items. According to this score, we classified the papers in three groups (Good, Fair, Poor) [6]. Data was extracted by two reviewers as described above. Dataincluded general information of studies, study eligibility, method, risk of bias assessment and results including odds ratio. If there were some unclear information, we contacted with corresponding authors of studies. Our data extraction form includes the following items: First author, Publication year, Source of study participants, Name of Country, Ethnicity, Genotyping method and Reference number. Association between the mentioned polymorphism and breast cancer was evaluated by calculating crude OR based on 2-by-2 table. Furthermore, this association was assessed after controlling potentially confounder variables. For this reason, we extracted adjusted OR values which were calculated by logistic regression in primary studies. Since multi-variable logistic regression models in primary studies were not similar, all adjusted OR values were extracted from primary studies in order to combine similar adjusted OR values in data synthesis step.

### Data synthesis (meta-analysis)

All of data analyses were performed in two distinct groups of familial breast cancer and sporadic breast cancer. Data were analyzed using STATA 13 software. Association between the mentioned SNP and breast cancer risk were analyzed by pooling odds ratio (ORs) with 95% confidence interval (CIs) in three models including dominant (TM + MM vs.TT), recessive (MM vs. TM + TT), and homozygote (MM vs.TT) models using STATA metan module. *Z* test was applied to assess the significance of the ORs, The heterogeneity between included publications was evaluated using I^2^ parameter as described previously [14] where the higher values indicate higher level of heterogeneity. Furthermore, we checked heterogeneity by the chi-square-based Q-test (Heterogeneity was considered statistically significant if *p* < 0.05) (Egger et al., 1997). We combined genotype frequencies to calculate univariable (crude) OR. In addition, combination of adjusted OR values was based on the similarity of adjusted OR values restricted in two models including age-adjusted (association between rs861539 and breast cancer after controlling age of patients) and age and other factors. The random-effects model was used to combine parameters acquired from discrete studies due to methodological variation. Sensitivity analyses were performed using leave-one-out sensitivity analysis to indicate the effect of the quality score on the results. Subgroup analyses were done for evaluating potential sources of heterogeneity based on ethnicity, case selection methods case group (hospital vs. population), methodological quality status (Good, Fair, Poor) and-case enrollment strategies (incident vs. prevalent).

### Publication bias

Funnel plots, Begg’s and Egger’s test were used to measure publication bias (*p*-value< 0.1) [6, 11].

## Results

### Literature search

**Fig. 1:**
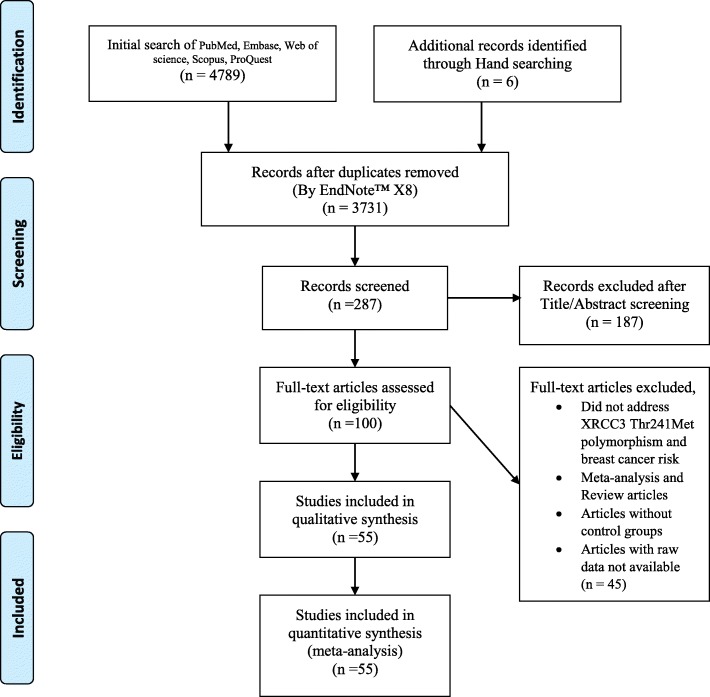
PRISMA flow diagram showing the selection of the 55 eligible case control studies

Figure 1 shows the data collection flow diagram for the present study. At the first step of database search, 4795 items were obtained. The initial screening and removal of duplicate items led to identification of 287 publications. Further screening resulted in removal of 187 items. Finally, full texts of the remained items were assessed for eligibility and 55 publications containing 30,966 sporadic breast cancer cases, 1174 familial breast cancer cases and 32,890 controls were included in the syntheses [8, 15–57]. Tables 1 and 2 show the features of selected studies which assessed the association between the mentioned SNP and breast cancer in familial and sporadic cases respectively.

**Table 1:** General characteristics of studies reporting associations in familial breast cancer (HB: hospital based, PB: population based, N/M: Not mentioned, HWE: Hardy-Weinberg Equilibrium, NOS: The Newcastle-Ottawa Scale, Quality of studies based on NOS star scoring system: 1–2 stars: poor, 3–5 stars: fair and 6–10 stars: good)

First Author	Year	Society	Country	Ethnicity	Genotyping Method	Case-enrollment strategy	Frequency in Cases				Frequency in Controls				HWE	NOS score
							TT	TM	MM	Total	TT	TM	MM	Total		
Costa	2007	HB	Portugal	Caucasian	PCR-RFLP	Prevalent	40	29	12	81	225	140	66	431	0	5
Dufloth	2005	HB	Brazil	Mixed	PCR-RFLP	Prevalent	27	18	7	52	68	35	15	118	0.005	3
Figueiredo	2004	PB	Canada	Caucasian	MALDI-TOF MS	Incident	29	38	16	83	13	20	4	37	0.341	9
Forsti	2004	PB	Finland	Caucasian	PCR-RFLP	Prevalent	72	85	15	172	89	88	25	202	0.654	4
Smith b	2003	HB	USA	Caucasian	PCR-RFLP	Incident	10	14	3	27	42	55	24	121	> 0.05	7
Vral	2011	HB	Italy	Caucasian	PCR-RFLP or SnapShot technique	N/M	60	87	23	170	54	84	30	168	0.964	2
Gonzalez-Hormazabal	2012	PB	Chile	Mixed	Taq-Man	Prevalent	187	103	32	322	335	209	23	567	0.177	7
Jara	2010	PB	Chile	Mixed	Conformation-sensitive gel electrophoresis (CSGE)	Prevalent	149	91	27	267	296	182	22	500	0.52	8

**Table 2:** General characteristics of studies reporting associations in sporadic breast cancer (HB: hospital based, PB: population based, HWE: Hardy-Weinberg Equilibrium, NOS: The Newcastle-Ottawa Scale)

First Author	Year	Society	Country	Ethnicity	Genotyping Method	Case-enrollment strategy	Frequency in Cases				Frequency in Controls				HWE	NOS Score
							TT	TM	MM	Total	TT	TM	MM	Total		
Al Zoubi	2015	HB	Jordan	Arab	Sequencing	Prevalent	16	26	4	46	8	18	5	31	0.33	5
Al Zoubi	2017	HB	Italy	Caucasian	Sequencing	Prevalent	8	13	2	23	4	9	2	15	0.72	5
Ali	2016	HB	Saudi Arabian	Arab	PCR-RFLP	Incident	43	73	27	143	32	32	78	35	> 0.05	6
Brooks	2008	PB	USA	Mixed	PCR-RFLP	Incident	254	259	98	611	249	286	76	611	0.661	9
Costa	2007	HB	Portugal	Caucasian	PCR-RFLP	Prevalent	68	77	31	176	121	61	29	211	0	5
Devi	2017	HB	India	Asian	PCR-RFLP	Prevalent	350	100	14	464	426	99	9	534	0.25	10
Ding	2015	HB	China	Asian	PCR-LDR	Prevalent	510	91	5	606	557	74	2	633	0.25	7
Dufloth	2005	HB	Brazil	Mixed	PCR-RFLP	Prevalent	15	16	2	33	68	35	15	118	0.005	3
Figueiredo	2004	PB	Canada	Caucasian	MALDI-TOF MS	incident	110	148	61	319	133	180	52	365	0.39	9
Forsti	2004	PB	Finland	Caucasian	PCR-RFLP	Prevalent	111	80	32	223	161	110	27	298	0.654	4
Garcia-Closas	2006	PB	Poland	Caucasian	NA	Incident	785	907	282	1974	980	1039	266	2285	0.709	7
Garcia-Closas	2006	PB	USA	Caucasian	NA	Incident	1102	1419	457	2978	973	1213	368	2554	0.748	7
Gohari-Lasaki	2015	HB	Iran	Mixed	PCR-RFLP	Prevalent	70	13	17	100	69	22	9	100	NA	2
Han	2004	PB	USA	Mixed	Taq-Man	Incident	388	429	135	952	468	607	170	1245	0.225	8
Jacobsen	2003	PB	Denmark	Caucasian	Taq-Man / PCR-RFLP	Incident	163	203	59	425	160	198	65	423	0.772	4
Kipen	2017	HB	Belarus	Caucasian	PCR-RFLP	Incident	86	68	15	169	84	94	7	185	> 0.05	5
Krupa	2009	HB	Poland	Caucasian	PCR-RFLP	Prevalent	29	68	38	135	29	107	39	175	0.003	4
Kuschel	2002	PB	UK	Caucasian	Taq-Man	Incident	790	1026	327	2143	728	827	229	1784	0.8	4
Lavanya	2015	HB	India	Asian	PCR-RFLP	N/M	42	7	1	50	40	8	2	50	> 0.05	6
Lee	2007	HB	South Korea	Asian	Single base extension assay	Prevalent	437	51	1	489	349	29	0	378	0.74	6
Loizidou	2008	PB	Cyprus	Mixed	PCR-RFLP	Incident	312	560	220	1092	351	600	226	1177	0.285	8
Millikan	2005	PB	USA	Caucasian	Taq-Man	Incident	505	578	171	1254	435	555	142	1132	0.086	9
Millikan	2005	PB	USA	African-American	Taq-Man	Incident	482	222	41	745	421	211	44	676	0.015	9
Ozgoz	2017	HB	Turkey	Mixed	Multiplex-PCR & MALDI-TOF	Prevalent	42	46	14	102	37	40	23	100	0.234	7
Qureshi	2014	HB	Pakistan	Mixed	PCR-RFLP	Prevalent	74	67	15	156	101	44	5	105	> 0.05	6
Rafii	2003	HB	UK	Caucasian	Taq-Man	Prevalent	201	248	72	521	341	416	129	886	0.87	8
Ramadan	2014	HB	Egypt	Mixed	PCR-RFLP	Incident	28	57	15	100	30	37	8	75	0.491	7
Romanowicz	2017	HB	Poland	Caucasian	HRM	Prevalent	48	72	80	200	52	72	76	200	0.862	6
Romanowicz-Makowska	2012	HB	Poland	Caucasian	PCR-RFLP	Prevalent	210	370	180	760	178	366	216	760	0.343	5
Romanowicz-Makowska	2011	HB	Poland	Caucasian	PCR-RFLP	Prevalent	220	378	192	790	188	384	226	798	0.939	5
Sangrajrang	2007	HB	Thai	Asian	Melting curve analysis	Incident	437	69	1	507	384	38	2	424	0.322	6
Santos	2010	HB	Brazil	Mixed	PCR-RFLP	Incident	28	31	6	65	49	29	7	85	0.37	6
Shadrina	2016	PB	Russia	Caucasian	Taq-Man	Prevalent	285	284	95	664	294	278	72	644	0.59	6
Silva	2010	HB	Portugal	Caucasian	PCR-RFLP	N/M	109	138	42	289	178	276	94	548	0.46	6
Smith	2008	HB	USA	Caucasian	Mass ARRAY system	Incident	124	137	54	315	158	184	59	401	0.649	5
Smith	2008	HB	USA	African-American	Mass ARRAY system	Incident	32	19	1	52	48	20	5	73	0.169	7
Smith a	2003	HB	USA	Caucasian	PCR-RFLP	Incident	96	105	51	252	104	129	35	268	0.611	7
Smith b	2003	PB	USA	Caucasian	PCR-RFLP	Incident	30	40	17	87	39	55	15	109	0.68	7
Smolarz	2015	HB	Poland	Caucasian	PCR-RFLP	Prevalent	19	35	16	70	15	35	20	70	0.718	6
Sobczuk	2009	HB	Poland	Caucasian	PCR-RFLP	Prevalent	29	71	50	150	24	50	32	106	0.567	5
Sterpone	2010	HB	Italy	Caucasian	PCR-RFLP	Prevalent	18	21	4	43	15	15	4	34	0.853	6
Su	2015	HB	Taiwan	Asian	PCR-RFLP	Prevalent	1052	141	39	1232	1131	87	14	1232	0.89	7
Thyagarajan	2006	PB	USA	Caucasian	PCR-RFLP	N/M	160	192	67	419	126	157	40	323	0.405	8
Vral	2011	HB	Italy	Caucasian	PCR-RFLP or SnapShot	N/M	13	22	9	44	54	84	30	168	0.964	2
Webb	2005	PB	Australia	Caucasian	Taq-Man	Prevalent	500	612	184	1296	248	321	91	660	0.425	8
Webb	2005	PB	Australia	Mixed	Taq-Man	Prevalent	91	44	14	149	59	54	15	128	0.625	8
Zhang	2005	HB	China	Asian	PCR-RFLP	Incident	33	80	107	220	29	115	166	310	0.17	3
BCAC HBBCS	2006	HB	Germany	Caucasian	Taq-Man & ARMS	N/M	95	119	42	1156	77	88	29	194	0.64	5
BCAC Madrid	2006	HB	Spain	Caucasian	Taq-Man & Illumina	N/M	255	274	92	621	281	287	105	673	0.028	6
BCAC SEARCH	2006	PB	UK	Caucasian	Taq-Man	N/M	1177	1462	465	3104	1607	1898	549	4054	0.76	9
BCAC Seoul	2006	HB	Korea	Asian	Taq-Man & SNPstream	N/M	502	53	1	556	355	31	0	386	0.411	8
BCAC Sheffield	2006	HB	UK	Caucasian	Taq-Man	N/M	458	555	168	1181	437	534	195	1166	0.144	7
BCAC USRTS	2006	PB	USA	Caucasian	Taq-Man	N/M	281	336	98	715	402	480	155	1037	0.55	7

### Meta-analysis results

Initially, we conducted the analysis in the familial and sporadic studies after using the random-effects model. Random model was used for analysis of associations in three inheritance models based on its more conservative nature. Final results for familial and sporadic studies are shown in Tables 3 and 4.

**Table 3:** Meta-analysis of studies reporting sporadic cases in different subgroups

Potential		Odd Ratio (CI 95%)	No of Studies	Heterogeneity χ^2^	P value	I^2^	Interaction p value
A Homozygote model: MM vs. TT							
Ethnicity	Caucasian	0.922 (0.838–1.016)	31	63.02	0.000	52.4%	0.0001
	Asian	0.725 (0.345–1.522)	8	18.89	0.009	62.9%	
	African-American	1.278 (0.826–1.977)	2	0.77	0.381	0.0%	
	Arab	3.649 (2.029–6.563)	2	0.26	0.609	0.0%	
	Mixed	0.889 (0.694–1.140)	10	16.49	0.009	45.4%	
Study-based	Hospital-based	0.979 (0.825–1.162)	36	81.66	0.000	57.1%	0.655
	Population-based	0.869 (0.796–0.950)	17	26.22	0.051	39.0%	
Methodological quality	Good	0.974 (0.786–1.208)	15	36.70	0.001	61.9%	0.891
	Fair	0.930 (0.830–1.041)	36	84.07	0.000	58.4%	
	Poor	0.644 (0.338–1.229)	2	0.37	0.544	0.0%	
Case enrollment strategies	Incident	0.938 (0.819–1.075)	20	54.88	0.000	59.9%	0.455
	Prevalent	0.887 (0.720–1.093)	23	45.70	0.001	58.4%	
	Not mentioned	0.975 (0.798–1.191)	10	21.53	0.011	58.2%	
All studies		0.937 (0.849–1.034)	53	124.20	0.000	58.1%	–
B Dominant model: TM + MM vs. TT							
Ethnicity	Caucasian	1.022 (0.969–1.079)	31	43.65	0.051	31.3%	0.0001
	Asian	1.296 (1.027–1.636)	8	18.22	0.011	61.6%	
	African-American	0.921 (0.749–1.134)	2	0.53	0.465	0.0%	
	Arab	0.671 (0.419–1.074)	2	0.00	0.950	0.0%	
	Mixed	1.084 (0.863–1.361)	10	33.91	0.000	73.5%	
Study-based	Hospital-based	1.089 (0.975–1.215)	36	89.81	0.000	61.0%	0.655
	Population-based	1.017 (0.955–1.084)	17	31.38	0.012	49.0%	
Methodological quality	Good	1.028 (0.950–1.112)	15	36.88	0.001	62.0%	0.891
	Fair	1.050 (1.010–1.091)	36	84.16	0.000	58.4%	
	Poor	1.022 (0.643–1.624)	2	0.12	0.725	0.0%	
Case enrollment strategies	Incident	1.011 (0.934–1.095)	20	37.53	0.007	49.4%	0.455
	Prevalent	1.111 (0.958–1.289)	23	74.40	0.000	70.4%	
	Not mentioned	1.042 (0.975–1.113)	10	7.89	0.545	0.0%	
All studies		1.045 (0.982–1.112)	53	121.39	0.000	57.2%	–
C Recessive model: MM vs. TM + TT							
Ethnicity	Caucasian	0.921 (0.849–1.000)	31	56.42	0.002	46.8%	0.000
	Asian	0.688 (0.374–1.266)	8	15.51	0.030	54.9%	
	African-American	1.265 (0.778–2.055)	2	1.02	0.312	2.2%	
	Arab	3.649 (2.029–6.563)	2	1.55	0.213	35.4%	
	Mixed	0.895 (0.728–1.101)	10	13.93	0.125	35.4%	
Study-based	Hospital-based	0.989 (0.844–1.159)	36	90.43	0.000	61.3%	0.00
	Population-based	0.868 (0.806–0.934)	17	21.79	0.150	26.6%	
Methodological quality	Good	0.961 (0.822–1.125)	15	27.19	0.018	48.5%	0.153
	Fair	0.942 (0.841–1.055)	36	99.37	0.000	64.8%	
	Poor	0.645 (0.355–1.173)	2	0.84	0.359	0.0%	
Case enrollment strategies	Incident	0.950 (0.823–1.097)	20	63.03	0.000	69.9%	0.377
	Prevalent	0.900 (0.761–1.064)	23	45.19	0.003	51.3%	
	Not mentioned	0.974 (0.812–1.168)	10	21	0.013	57.1%	
All studies		0.939 (0.857–1.029)	55	131.15	0.000	60.3%	–

**Table 4:** Meta-analysis of studies reporting familial cases in different subgroups

Potential		Odd Ratio (CI 95%)	No of Studies	Heterogeneity χ^2^	*P* value	I^2^	Interaction *p* value
A Homozygote model: MM vs. TT							
Ethnicity	Caucasian	1.204 (0.835–1.735)	5	2.56	0.634	0.0%	0.000
	Mixed	0.451 (0.309–0.659)	3	1.8	0.406	0.0%	
Study-based	Hospital-based	1.184 (0.784–1.788)	4	1.52	0.677	0.0%	0.690
	Population-based	0.581 (0.318–1.060)	4	8.24	0.041	63.6%	
Methodological quality	Good	1.080 (0.691–1.688)	3	0.67	0.716	0.0%	0.002
	Fair	0.504 (0.304–0.834)	4	4.51	0.211	33.5%	
	Poor	1.449 (0.752–2.793)	1	0.00	.	.%	
Case enrollment strategies	Incident	1.000 (0.300–3.327)	2	1.64	0.201	38.9%	0.068
	Prevalent	0.683 (0.412–1.134)	5	10.69	0.030	62.6%	
	Not mentioned	1.449 (0.752–2.793)	1	0	.	.%	
All studies		0.809 (0.521–1.258)	8	17.7	0.013	60.4%	–
B Dominant model: TM + MM vs. TT							
Ethnicity	Caucasian	1.012 (0.800–1.280)	5	0.82	0.936	0.0%	0.576
	Mixed	1.104 (0.909–1.341)	3	0.39	0.824	0.0%	
Study-based	Hospital-based	1.016 (0.770–1.341)	4	1.11	0.775	0.0%	0.690
	Population-based	1.087 (0.910–1.299)	4	0.25	0.969	0.0%	
Methodological quality	Good	1.132 (0.855–1.499)	3	0.13	0.937	0.0%	0.614
	Fair	1.075 (0.887–1.304)	4	0.41	. 0.937	0.0%	
	Poor	0.868 (0.553–1.364)	1	0.00	.	.%	
Case enrollment strategies	Incident	0.958 (0.530–1.733)	2	0.39	0.201	38.9%	0.579
	Prevalent	1.104 (0.936–1.302)	5	0.03	0.856	0.0%	
	Not mentioned	0.868 (0.553–1.364)	1	0	.	.%	
All studies		1.066 (0.917–1.238)	8	1.52	0.982	0.0%	–
C Recessive model: MM vs. TM + TT							
Ethnicity	Caucasian	1.233 (0.877–1.732)	5	**3.41**	0.491	0.0%	0.576
	Mixed	0.462 (0.298–0.716)	3	2.65	0.266	24.5%	
Study-based	Hospital-based	1.224 (0.834–1.796)	4	1.25	0.742	0.0%	0.690
	Population-based	0.409 (0.228–0.734)	4	10.89	0.012	72.4%	
Methodological quality	Good	1.172 (0.765–1.793)	3	0.79	0.675	0.0%	0.614
	Fair	0.515 (0.297–0.894)	4	5.63	0.131	46.7%	
	Poor	1.389 (0.770–2.508)	1	0.00	. 2.508	–	
Case enrollment strategies	Incident	0.977 (0.258–3.707)	5	14.05	0.007	71.5%	0.579
	Prevalent	0.718 (0.410–1.257)	2	2.36	0.124	57.7%	
	Not mentioned	1.389 (0.770–2.508)	1	0.00	–	–	
All studies		0.831 (0.524–1.319)	8	21.53	0.003	67.5%	–

Bold entry is significant

**Fig. 2:**
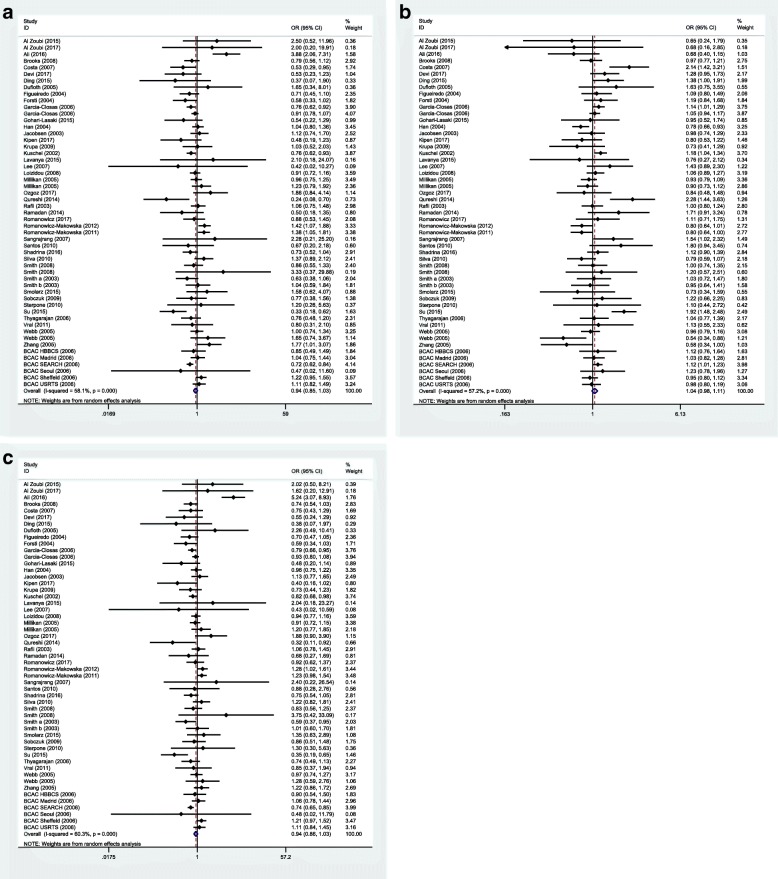
Forest plots of *XRCC3* Thr241Met polymorphism and sporadic breast cancer for all eligible studies. **a** Homozygote model: MM vs. TT. **b** Dominant model: TM + MM vs. TT. **c** Recessive model: MM vs. TM + TT

**Fig. 3:**
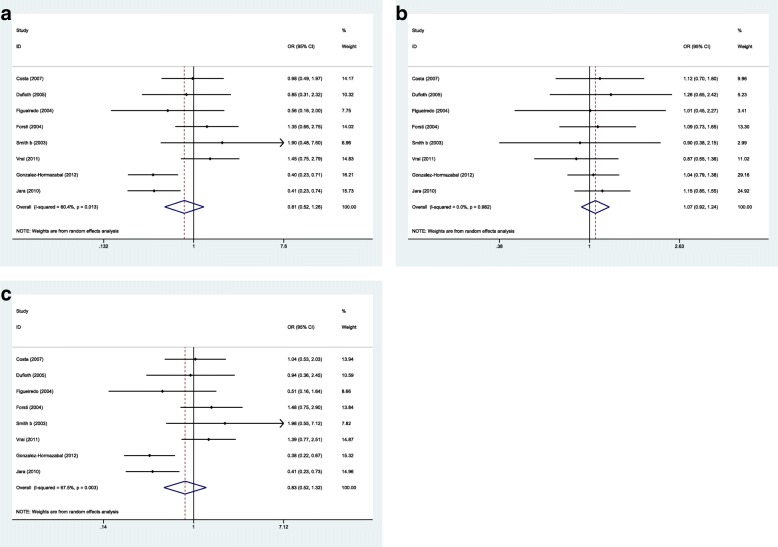
Forest plots of *XRCC3* Thr241Met polymorphism and familial breast cancer for all eligible studies. **a** Homozygote model: MM vs. TT. **b** Dominant model: TM + MM vs. TT. **c** Recessive model: MM vs. TM + TT

The forest plots for each model are depicted in Figs. 2 and 3.

No significant associations were detected between the mentioned SNP and breast cancer risk in any inheritance model either in familial or in sporadic breast cancer cases.

**Fig. 4:**
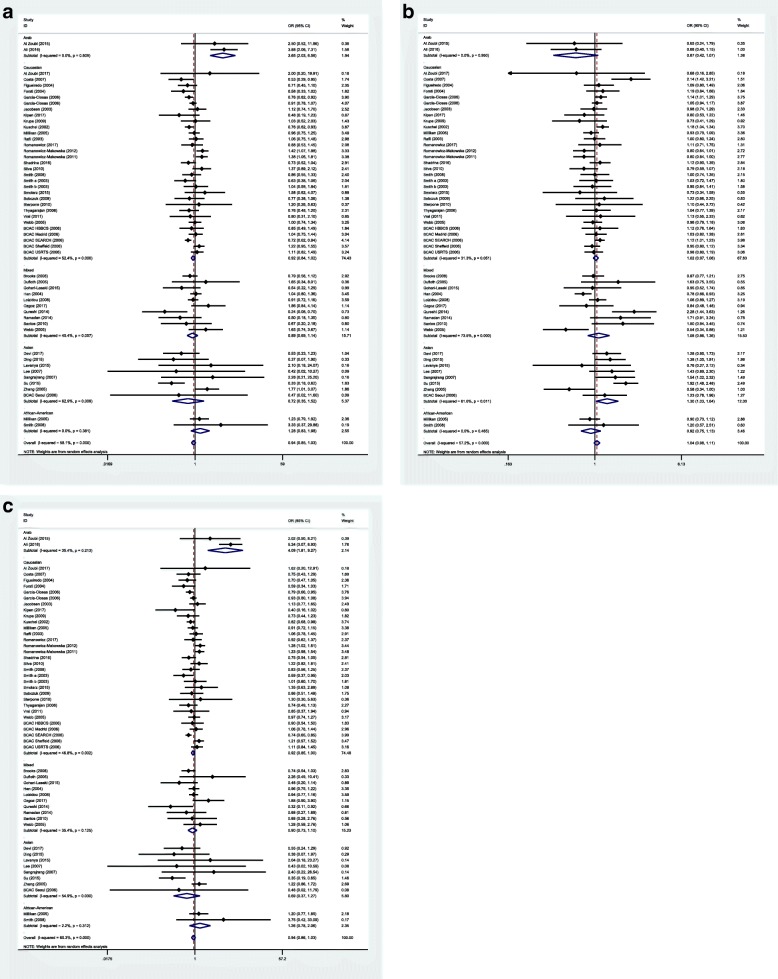
Forest plots of *XRCC3* Thr241Met polymorphism and risk of sporadic breast cancer in ethnic-based subgroups. **a** Homozygote model: MM vs. TT. **b** Dominant model: TM + MM vs. TT. **c** Recessive model: MM vs. TM + TT

**Fig. 5:**
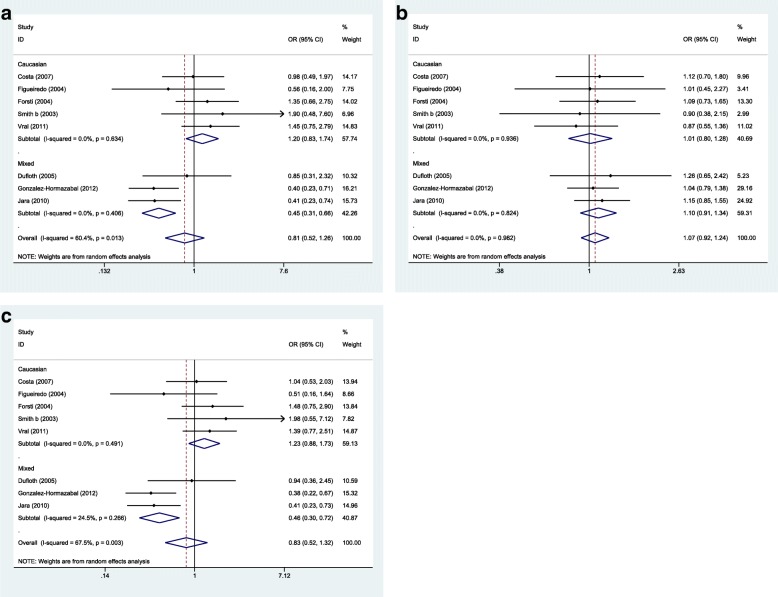
Forest plots of *XRCC3* Thr241Met polymorphism and risk of familial breast cancer in ethnic-based subgroups. **a** Homozygote model: MM vs. TT. **b** Dominant model: TM + MM vs. TT. **c** Recessive model: MM vs. TM + TT

Next, we assessed association between this SNP and risk of familial or sporadic breast cancer in ethnic-based subgroups (Figs. 4 and 5). In sporadic breast cancer, the SNP was associated with breast cancer risk in Arab populations in homozygous (OR (95% CI) = 3.649 (2.029–6.563), *p* = 0.0001) and recessive models (OR (95% CI) = 4.092 (1.806–9.271), *p* = 0.001). However, the association was significant in Asian population in dominant model (OR (95% CI) = 1.296 (1.027–1.636), *p* = 0.029). Based on the calculated Interaction *p*-value in ethnic-based subgroup analyses (p = 0.0001), we conclude that such subgroup analysis strategy was appropriate and the calculated ORs are significant. However, the associations was significant in familial breast cancer in mixed ethnic-based subgroup in homozygote and recessive models (OR (95% CI) = 0.451 (0.309–0.659), *p* = 0.0001, OR (95% CI) = 0.462 (0.298–0.716), *p* = 0.001 respectively).

Subsequently, we appraised the associations based on the study-base for selecting case/control (society) subgroup (hospital-based vs. population-based). In sporadic cases, the associations were significant in population-based studies in homozygote and recessive models (OR (95% CI) = 0.869 (0.796–0.950), *p* = 0.002 and OR (95% CI) = 0.868 (0.806–0.934), *p* = 0.0001 respectively). The Interaction *p*-value was calculated as 0.655 which shows inappropriateness of such subgroup analysis strategy. No significant associations were found in society-based analysis in familial cases (Additional file 2: Figure S1 and Additional file 3: Figure S2).

We also assessed the associations in methodological quality subgroups (Based on NOS scores) and found no significant association in sporadic (Interaction *p*-value = 0.891) but in familial cases we found the association in studies with fair quality in homozygote and recessive models (OR (95% CI) = 0.504 (0.304–0.834), *p* = 0.008, OR (95% CI) = 0.515 (0.297–0.894), *p* = 0.018 respectively) (Additional file 4: Figure S3 and Additional file 5: Figure S4).

Finally, we evaluated associations based on the case enrollment strategy (Incident vs. Prevalent). No significant associations were detected either in sporadic or familial cases (Interaction p-value = 0.22) (Additional file 6: Figure S5 and Additional file 7: Figure S6).

### Publication bias

**Fig. 6:**
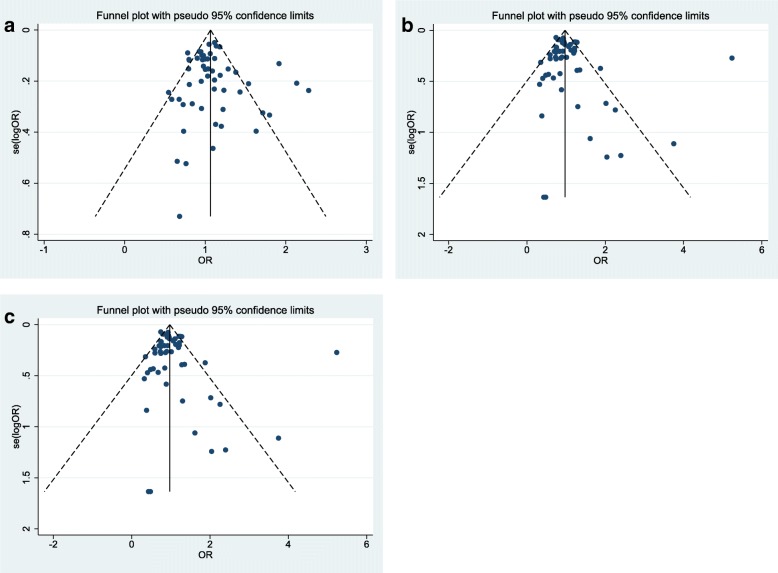
Funnel plots for whole publications in sporadic cases. **a** Dominant model: TM + MM vs.TT. **b** Recessive model: MM vs. TM + TT. **c** Homozygote model: MM vs.TT

**Fig. 7:**
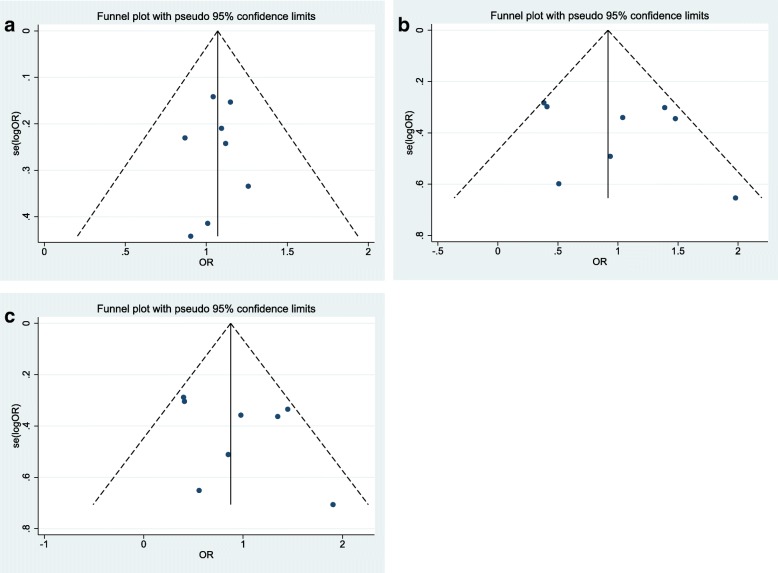
Funnel plots for whole publications in familial cases. **a** Dominant model: TM + MM vs.TT. **b** Recessive model: MM vs. TM + TT. **c** Homozygote model: MM vs.TT

We conducted both Begg’s funnel plot and Egger’s test for appraisal of the publication bias in sporadic and familial studies separately. The calculated parameters are shown in Tables 3 and 4. Moreover, the outlines of the funnel plots were rather symmetric implying absence of any significant publication bias (Figs. 6 and 7).

### Adjusted OR

**Fig. 8:**
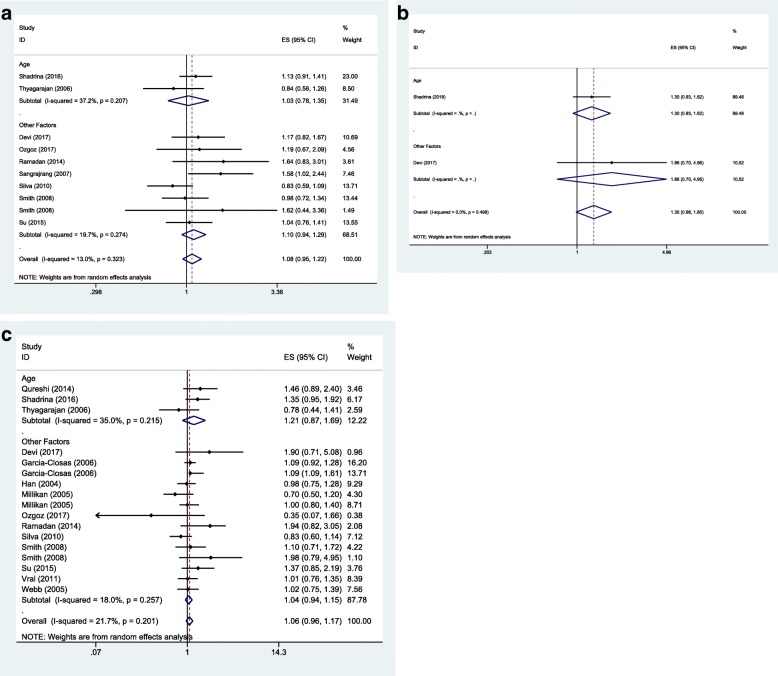
Forest plots for adjusted OR (adjusted for Age and Other variables including body mass index, smoking, hazardous life style and contraceptive use.) in sporadic cases. **a** Dominant model: TM + MM vs.TT. **b** Recessive model: MM vs. TM + TT. **c** Homozygote model: MM vs.TT

As we did not detected any association between the mentioned SNP and breast cancer risk in crude analysis, we subsequently assessed associations considering the effects of confounder variables using adjusted ORs. We retrieved adjusted ORs and confounder variables from the publications. Subsequently, we categorized confounder variables to two groups: 1. Age 2. Other variables including body mass index, smoking, hazardous life style and contraceptive use. Analyses were performed in sporadic subgroup based on the three inheritance models (Fig. 8). There was no significant association between this SNP and risk of sporadic breast cancer in any inheritance model considering adjusted ORs.

### Sensitivity analysis and cumulative meta-analysis

To assess the strength of the association results, we conducted a leave-one-out sensitivity analysis by repeatedly removing one study at a time and re-measuring the summary OR. The summary ORs did not change, showing that our results were not originated from any certain study (Table 1).

## Discussion

In the present meta-analysis, we assessed the associations between Thr241Met SNP and familial/ sporadic breast cancer based on the results of 55 studies containing 30,966 sporadic breast cancer cases, 1174 familial breast cancer cases and 32,890 controls. Crude analyses revealed no associations. In spite of assessing potential confounder variables and adjusting odds ratio of the primary studies, we did not find any association.

In sporadic cases, the narrow confidence intervals indicate the high power of the meta-analysis, so the results are conclusive. However, in familial cases, the wide confidence intervals imply that further studies are needed to reach conclusive results. Based on such findings, we predict that inclusion of further studies would not change the results of the meta-analysis. Sensitivity analyses by repeatedly removing one study at a time showed that the results of crude analysis were consistent result, therefore signifying the robustness of the study according to sensitivity analysis results, no relation between quality of studies with results and non-considerable publication bias.

Another strong point of our study was that we considered adjusted ORs to control the effects of confounding variables. Such approach further verified our results.

Through calculation of Interaction *p* values we determined subgroup analysis based on ethnicity as being the most strategy in this regard. Ethnic based analysis showed that in sporadic breast cancer, the SNP was associated with breast cancer risk in Arab and Mixed populations in homozygous and recessive models. The association was significant in Asian population in dominant model. However, no associations were detected in familial breast cancer in any ethnic-based subgroup and any inheritance model. The detected associations between this SNP and risk of sporadic breast cancer in certain populations had wide confidence intervals which necessitate extra studies. The same situation has been seen in familial breast cancer cases in ethnic-based subgroup analyses.

Chai et al. have performed a meta-analysis of 23 case-controls studies on association between Thr241Met SNP and breast cancer. Their meta-analysis of the pooled data of 13,513 cases and 14,100 controls association between the mentioned SNP and breast cancer risk in recessive and homozygote models in total populations as well as within Asian populations [14]. Our study had the advantage of including higher numbers of cases and controls and assessment of adjusted ORs and sensitivity analysis. The results of our ethnic-based analysis were consistent with their results regarding the observed association in Asian population but not regarding the associated model. Although they found association between this SNP and risk of sporadic breast cancer, we disapprove such association based on the obtained conclusive results.

In brief, we have implemented the high quality systematic review and meta-analysis including comprehensiveness (inclusion of 5 databases), inclusion of grey literature (theses) and duplicate implementation of all steps of systematic review and meta-analysis (independent implementation of search, screening, selection, quality assessment and data extraction by two authors). In addition, priori principle (establishment and registration of protocol) was applied.

Our study had some limitations. Based on the unavailability of sufficient data from the primary studies, we could not assess the association between the mentioned SNP and breast cancer risk in pre−/post-menopause subgroups. In addition, the adjusted OR values of the primary studies were based on different parameters which might influence the validity of this kind of statistical analysis. Finally, there were some limitations in the primary studies and we did not find any genotyping data according to breast cancer subtypes except for 3 studies in triple negative breast cancer. Due to the low number of primary studies, the result of meta-analysis based on breast cancer subtypes was not reliable. So, we did not performed this type of analysis.

## Conclusion

Taken together, our results in a large sample of both sporadic and familial cases of breast cancer showed insignificant role of Thr241Met in the pathogenesis of this type of malignancy. Such results were more conclusive in sporadic cases. In familial cases, future studies are needed to verify our results.

## Additional files

Additional file 1: The search syntaxes for each database. (DOCX 14 kb)

Additional file 2: **Figure S1.** Forest plots of *XRCC3* Thr241Met polymorphism and risk of sporadic breast cancer in Study-based subgroups. (D) Homozygote model: MM vs. TT. (E) Dominant model: TM + MM vs. TT. (F) Recessive model: MM vs. TM + TT. (ZIP 21 kb)

Additional file 3: **Figure S2.** Forest plots of *XRCC3* Thr241Met polymorphism and risk of familial breast cancer in society -based subgroups. (D) Homozygote model: MM vs. TT. (E) Dominant model: TM + MM vs. TT. (F) Recessive model: MM vs. TM + TT. (ZIP 8 kb)

Additional file 4: **Figure S3.** Forest plots of *XRCC3* T241 M Polymorphism and Sporadic Breast Cancer according to NOS subgroup analysis. (A) Homozygote model: MM vs. TT. (B) Dominant model: TM + MM vs. TT. (C) Recessive model: MM vs. TM + TT. (ZIP 22 kb)

Additional file 5: **Figure S4.** Forest plots of *XRCC3* T241 M Polymorphism and Familial Breast Cancer according to NOS subgroup analysis. (A) Homozygote model: MM vs. TT. (B) Dominant model: TM + MM vs. TT. (C) Recessive model: MM vs. TM + TT. (ZIP 9 kb)

Additional file 6: **Figure S5.** Forest plots of *XRCC3* T241 M Polymorphism and Sporadic Breast Cancer according to case enrollment subgroup analysis. (A) Homozygote model: MM vs. TT. (B) Dominant model: TM + MM vs. TT. (C) Recessive model: MM vs. TM + TT. (ZIP 22 kb)

Additional file 7: **Figure S6.** Forest plots of *XRCC3* T241 M Polymorphism and Familial Breast Cancer according to case enrollment subgroup analysis. (A) Homozygote model: MM vs. TT. (B) Dominant model: TM + MM vs. TT. (C) Recessive model: MM vs. TM + TT. (ZIP 9 kb)

## Abbreviations

CIs: 95% confidence intervals
DSB: DNA double strand break
HB: Hospital based
HR: Homologous recombination
HWE: Hardy-Weinberg Equilibrium
NHEJ: Non-homologous end-joining
NOS: Newcastle–Ottawa Scale
ORs: Crude odds ratios
PB: Population based
SNP: Single-nucleotide polymorphism
*XRCC3*: *X-ray repair cross-complementing group 3*
