# Supplementary material for: Associations between XRCC3 Thr241Met polymorphisms and breast cancer risk: systematic-review and meta-analysis of 55 case-control studies
Source: BMC Med Genet. 2019 May 10;20:79. doi: 10.1186/s12881-019-0809-8 (PMC6511159; doi:10.1186/s12881-019-0809-8)
Supplement: Supplementary file 1 — The search syntaxes for each database. (DOCX 14 kb) [file 12881_2019_809_MOESM1_ESM.docx]

Search syntax

PubMed

("Breast Neoplasm" OR (Neoplasm AND Breast) OR "Breast tumor" OR (Tumor AND Breast) OR "Breast Carcinoma" OR (Carcinoma AND Breast) OR "Breast Cancer" OR (Cancer AND Breast) OR "mamma* tumor" OR (mamma* AND tumor)) AND ("XRCC3 gene polymorphism" OR " XRCC3 gene single nucleotide polymorphism" OR " XRCC3 gene SNP" OR (XRCC3 AND polymorphism) OR (XRCC3 AND SNP) OR " XRCC3 variant" OR (XRCC3 AND variant) OR (x-ray repair cross-complementing group 3 AND polymorphism) OR (x-ray repair cross-complementing group 3 AND SNP) OR rs861539 OR c.722C>T OR p.Thr241Met OR T241M OR rs1799794 OR rs1799796 OR rs45603942 OR rs861530 OR rs3212057 OR rs28903081) AND (1990/01/01:2018/03/31[dp])

Scopus

(ALL("Breast Neoplasm") OR (ALL(Neoplasm) AND ALL(Breast)) OR ALL("Breast tumor") OR (ALL(Tumor) AND ALL(Breast)) OR ALL(" Breast Carcinoma") OR (ALL(Carcinoma) AND ALL(Breast)) OR ALL(" Breast Cancer") OR (ALL(Cancer) AND ALL(Breast)) OR ALL("mamma* tumor") OR (ALL(mamma*) AND ALL(tumor))) AND (ALL("XRCC3 gene polymorphism") OR ALL(" XRCC3 gene single nucleotide polymorphism") OR ALL(" XRCC3 gene SNP") OR (ALL(XRCC3) AND ALL(polymorphism)) OR (ALL(XRCC3) AND ALL(SNP)) OR ALL(" XRCC3 variant") OR (ALL(XRCC3) AND ALL(variant)) OR (ALL(“x-ray repair cross-complementing group 3”) AND ALL(polymorphism)) OR (ALL(“x-ray repair cross-complementing group 3”) AND ALL(SNP)) OR ALL(rs861539) OR ALL(c.722C>T) OR ALL(p.Thr241Met) OR ALL(T241M) OR ALL(rs1799794) OR ALL(rs1799796) OR ALL(rs45603942) OR ALL(rs861530) OR ALL(rs861539) OR ALL(rs3212057) OR ALL(rs28903081)) AND ((PUBYEAR>1990 AND PUBYEAR<2018) AND (PUBDATETXT(January2018) OR PUBDATETXT(February2018) OR PUBDATETXT(March2018)))

EMBASE

('breast neoplasm' OR (('neoplasm'/exp OR neoplasm) AND ('breast'/exp OR breast)) OR 'breast tumor'/exp OR 'breast tumor' OR (('tumor'/exp OR tumor) AND ('breast'/exp OR breast)) OR 'breast carcinoma'/exp OR 'breast carcinoma' OR (('carcinoma'/exp OR carcinoma) AND ('breast'/exp OR breast)) OR 'breast cancer'/exp OR 'breast cancer' OR (('cancer'/exp OR cancer) AND ('breast'/exp OR breast)) OR 'mamma* tumor' OR (mamma* AND ('tumor'/exp OR tumor))) AND ('xrcc3 gene polymorphism' OR 'xrcc3 gene single nucleotide polymorphism' OR 'xrcc3 gene snp' OR (xrcc3 AND ('polymorphism'/exp OR polymorphism)) OR (xrcc3 AND ('snp'/exp OR snp)) OR 'xrcc3 variant' OR (xrcc3 AND variant) OR ('x-ray repair cross-complementing group 3' AND ('polymorphism'/exp OR polymorphism)) OR ('x-ray repair cross-complementing group 3' AND ('snp'/exp OR snp)) OR rs861539 OR 'c.722c-t' OR 'p.thr241met' OR 't241m' OR 'rs1799794' OR 'rs1799796' OR 'rs45603942' OR 'rs861530' OR 'rs3212057' OR 'rs2890308') AND [1990-2019]/py

Web of science

(TS=("Breast Neoplasm") OR (TS=(Neoplasm) AND TS=(Breast)) OR TS=("Breast tumor") OR (TS=(Tumor) AND TS=(Breast)) OR TS=("Breast Carcinoma") OR (TS=(Carcinoma) AND TS=(Breast)) OR TS=("Breast Cancer") OR (TS=(Cancer) AND TS=(Breast)) OR TS=(" mamma* tumor") OR (TS=(mamma*) AND TS=(tumor))) AND (TS=("XRCC3 gene polymorphism") OR TS=("XRCC3 gene single nucleotide polymorphism") OR TS=(" XRCC3 gene SNP") OR (TS=(XRCC3) AND TS=(polymorphism)) OR (TS=(XRCC3) AND TS=(SNP)) OR TS=(" XRCC3 variant") OR (TS=(XRCC3) AND TS=(variant)) OR (TS=(“x-ray repair cross-complementing group 3”) AND TS=(polymorphism)) OR (TS=(“x-ray repair cross-complementing group 3”) AND TS=(SNP)) OR TS=(rs861539) OR TS=(c.722C>T) OR TS=(p.Thr241Met) OR TS=(T241M) OR TS=(rs1799794) OR TS=(rs1799796) OR TS=(rs45603942) OR TS=(rs861530) OR TS=(rs861539) OR TS=(rs3212057) OR TS=(rs28903081)) AND (PY=(1990-2019))

PROQUEST

(ALL,FT("Breast Neoplasm") OR (ALL,FT(Neoplasm) AND ALL,FT(Breast)) OR ALL,FT("Breast tumor") OR (ALL,FT(Tumor) AND ALL,FT(Breast)) OR ALL,FT(" Breast Carcinoma") OR (ALL,FT(Carcinoma) AND ALL,FT(Breast)) OR ALL,FT(" Breast Cancer") OR (ALL,FT(Cancer) AND ALL,FT(Breast)) OR ALL,FT("mamma* tumor") OR (ALL,FT(mamma*) AND ALL,FT(tumor))) AND (ALL,FT("XRCC3 gene polymorphism") OR ALL,FT(" XRCC3 gene single nucleotide polymorphism") OR ALL,FT(" XRCC3 gene SNP") OR (ALL,FT(XRCC3) AND ALL,FT(polymorphism)) OR (ALL,FT(XRCC3) AND ALL,FT(SNP)) OR ALL,FT(" XRCC3 variant") OR (ALL,FT(XRCC3) AND ALL,FT(variant)) OR (ALL,FT(“x-ray repair cross-complementing group 3”) AND ALL,FT(polymorphism)) OR (ALL,FT(“x-ray repair cross-complementing group 3”) AND ALL,FT(SNP)) OR ALL,FT(rs861539) OR ALL,FT(c.722C>T) OR ALL,FT(p.Thr241Met) OR ALL,FT(T241M) OR ALL,FT(rs1799794) OR ALL,FT(rs1799796) OR ALL,FT(rs45603942) OR ALL,FT(rs861530) OR ALL,FT(rs861539) OR ALL,FT(rs3212057) OR ALL,FT(rs28903081)) AND (PD(19900101-20180331))
