# Supplementary figures and images for: Associations between XRCC3 Thr241Met polymorphisms and breast cancer risk: systematic-review and meta-analysis of 55 case-control studies
Source: BMC Med Genet. 2019 May 10;20:79. doi: 10.1186/s12881-019-0809-8 (PMC6511159; doi:10.1186/s12881-019-0809-8)

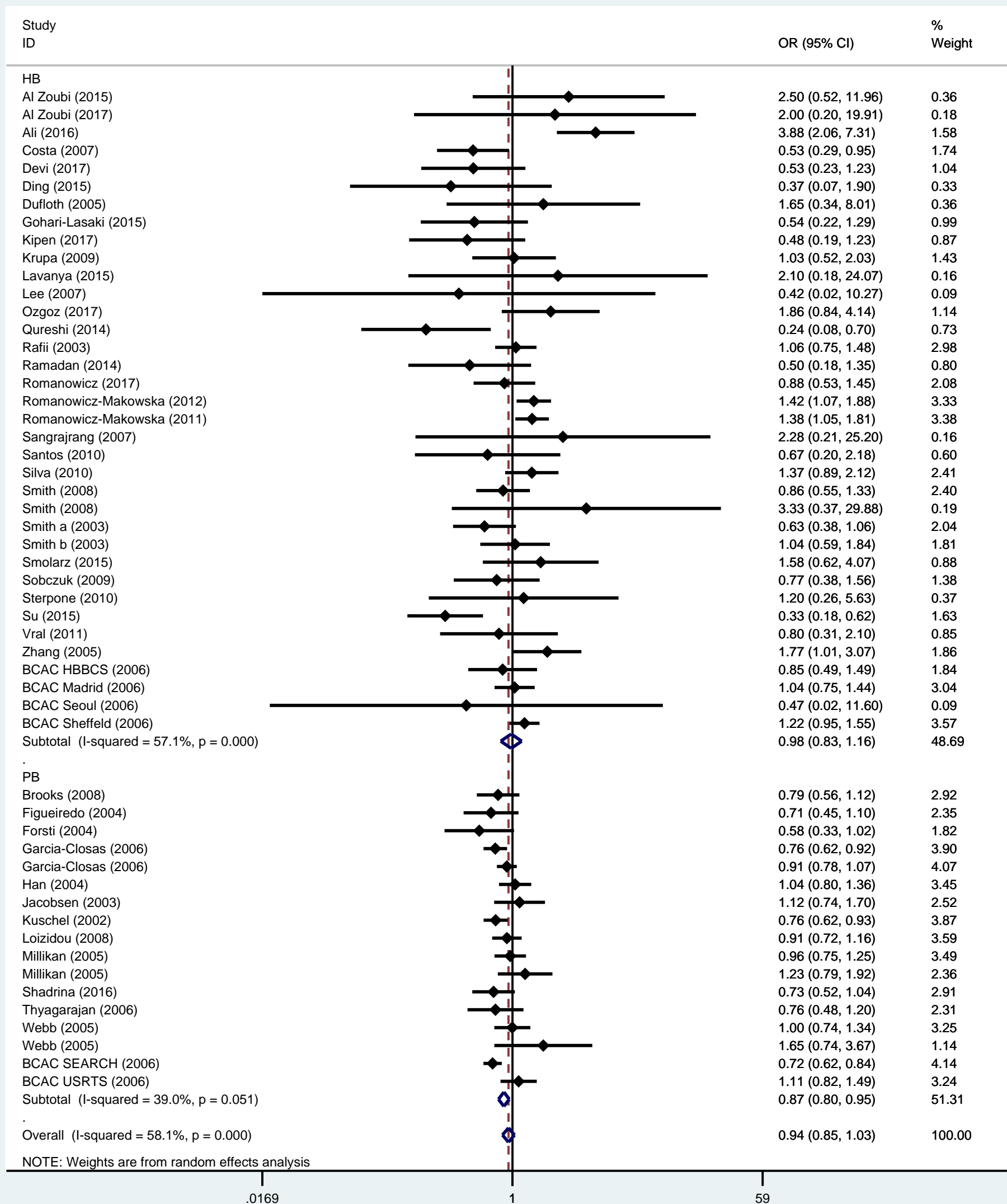

Supplement: Supplementary file 2 — Figure S1. Forest plots of XRCC3 Thr241Met polymorphism and risk of sporadic breast cancer in Study-based subgroups. (D) Homozygote model: MM vs. TT. (E) Dominant model: TM + MM vs. TT. (F) Recessive model: MM vs. TM + TT. (ZIP 21 kb) [file 12881_2019_809_MOESM2_ESM.zip › Figure S1 AR3.pdf]

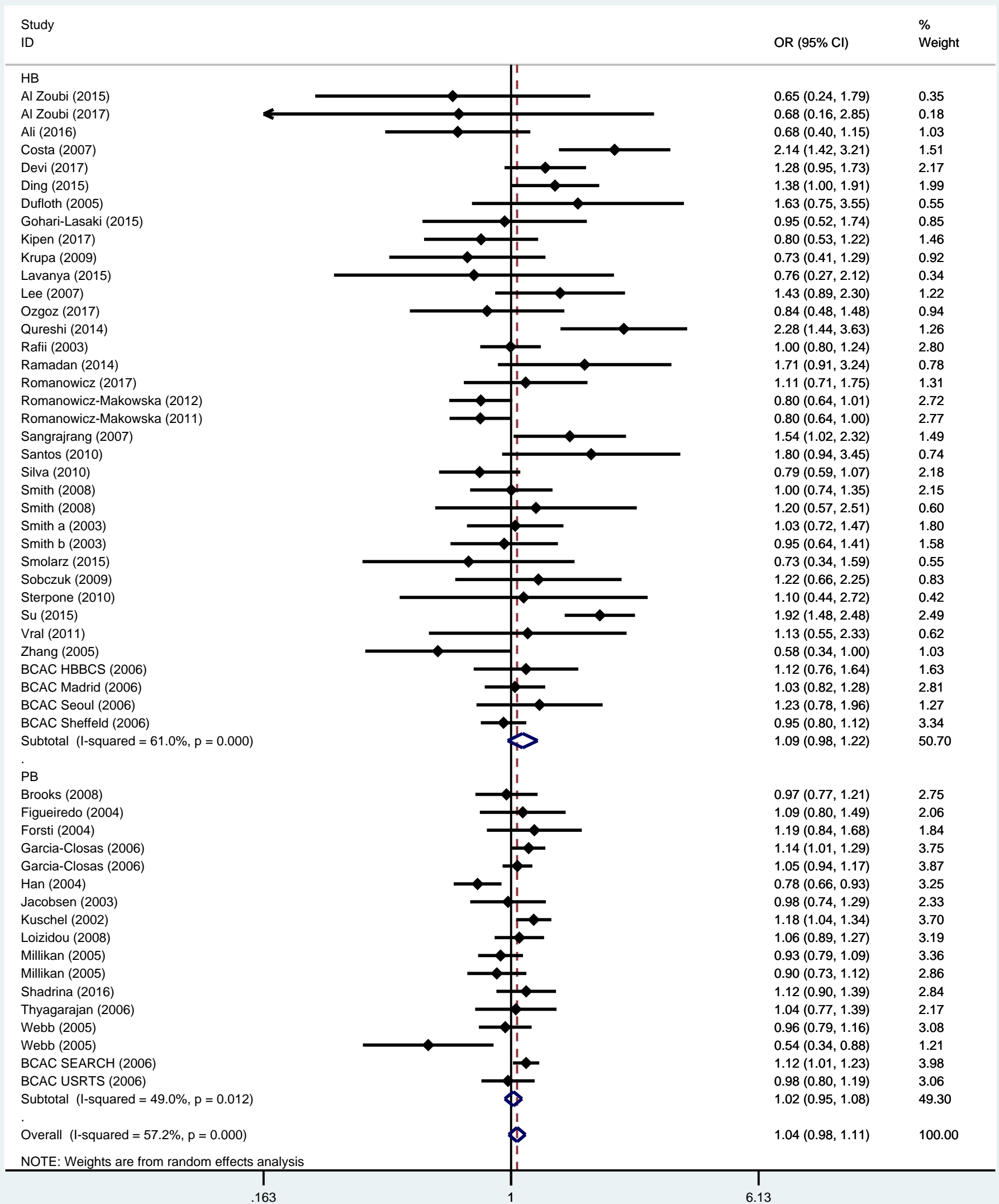

Supplement: Supplementary file 2 — Figure S1. Forest plots of XRCC3 Thr241Met polymorphism and risk of sporadic breast cancer in Study-based subgroups. (D) Homozygote model: MM vs. TT. (E) Dominant model: TM + MM vs. TT. (F) Recessive model: MM vs. TM + TT. (ZIP 21 kb) [file 12881_2019_809_MOESM2_ESM.zip › Figure S1 BR3.pdf]

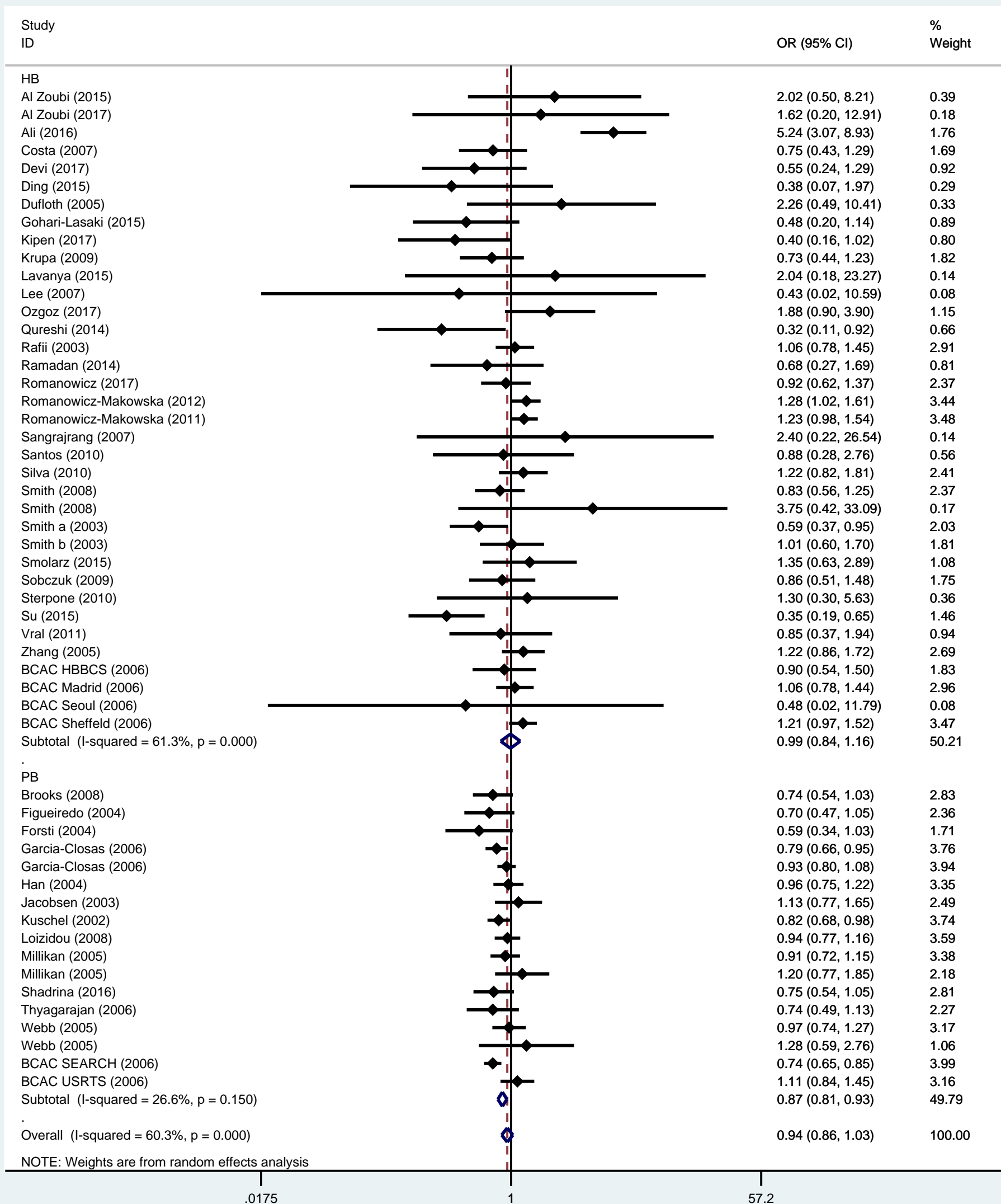

Supplement: Supplementary file 2 — Figure S1. Forest plots of XRCC3 Thr241Met polymorphism and risk of sporadic breast cancer in Study-based subgroups. (D) Homozygote model: MM vs. TT. (E) Dominant model: TM + MM vs. TT. (F) Recessive model: MM vs. TM + TT. (ZIP 21 kb) [file 12881_2019_809_MOESM2_ESM.zip › Figure S1 CR3.pdf]

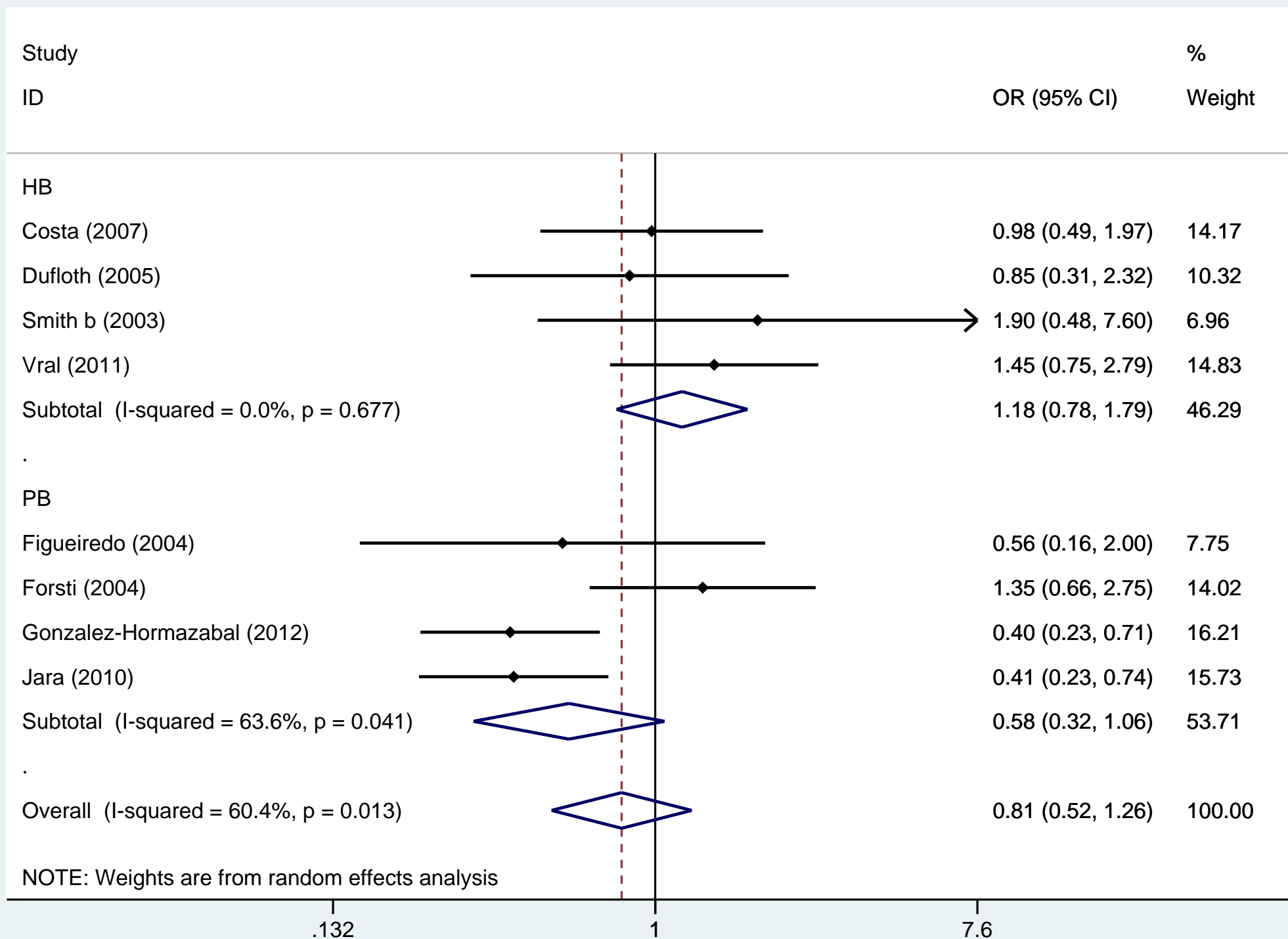

Supplement: Supplementary file 3 — Figure S2. Forest plots of XRCC3 Thr241Met polymorphism and risk of familial breast cancer in society -based subgroups. (D) Homozygote model: MM vs. TT. (E) Dominant model: TM + MM vs. TT. (F) Recessive model: MM vs. TM + TT. (ZIP 8 kb) [file 12881_2019_809_MOESM3_ESM.zip › Figure S2 AR3.pdf]

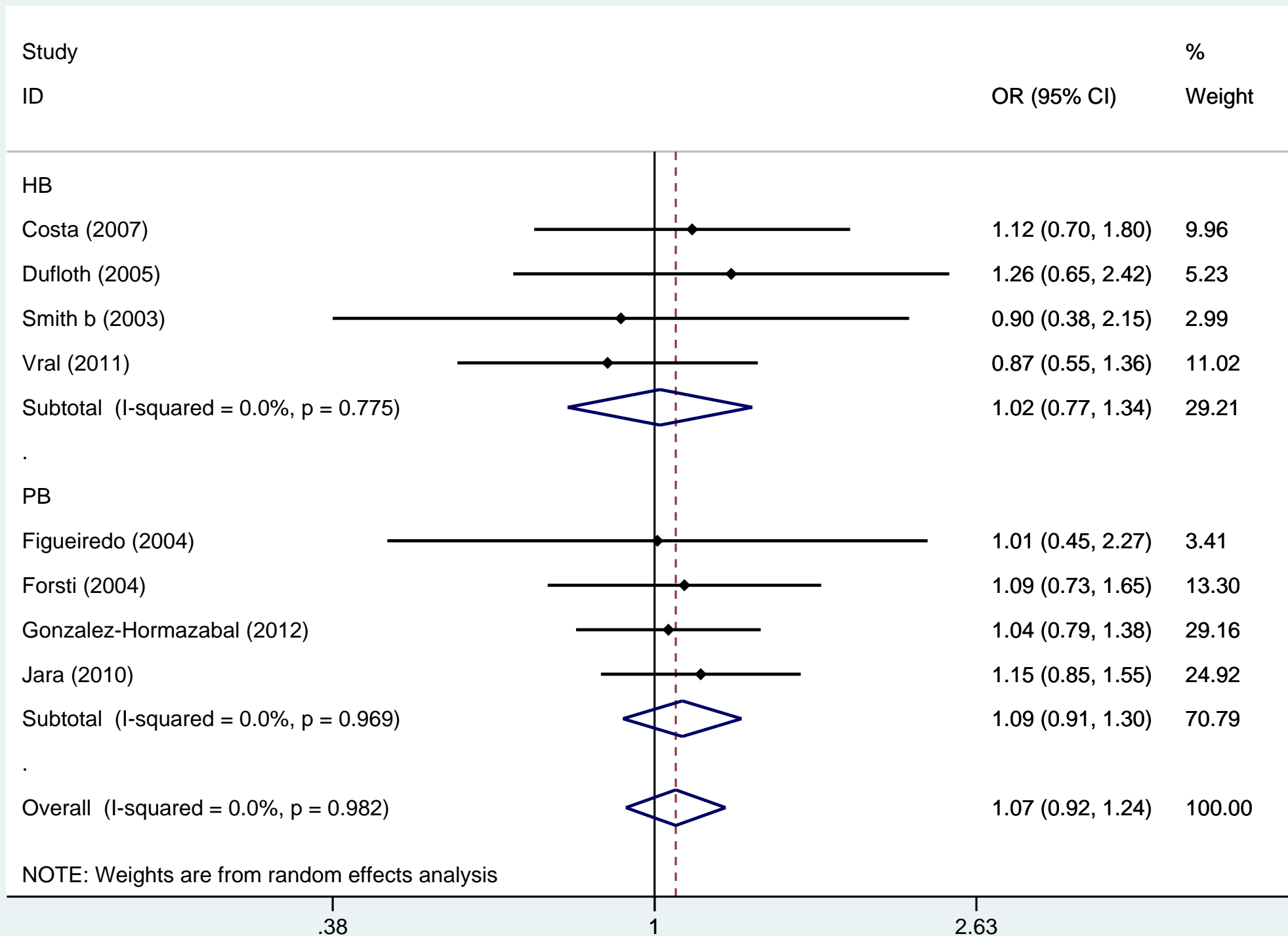

Supplement: Supplementary file 3 — Figure S2. Forest plots of XRCC3 Thr241Met polymorphism and risk of familial breast cancer in society -based subgroups. (D) Homozygote model: MM vs. TT. (E) Dominant model: TM + MM vs. TT. (F) Recessive model: MM vs. TM + TT. (ZIP 8 kb) [file 12881_2019_809_MOESM3_ESM.zip › Figure S2 BR3.pdf]

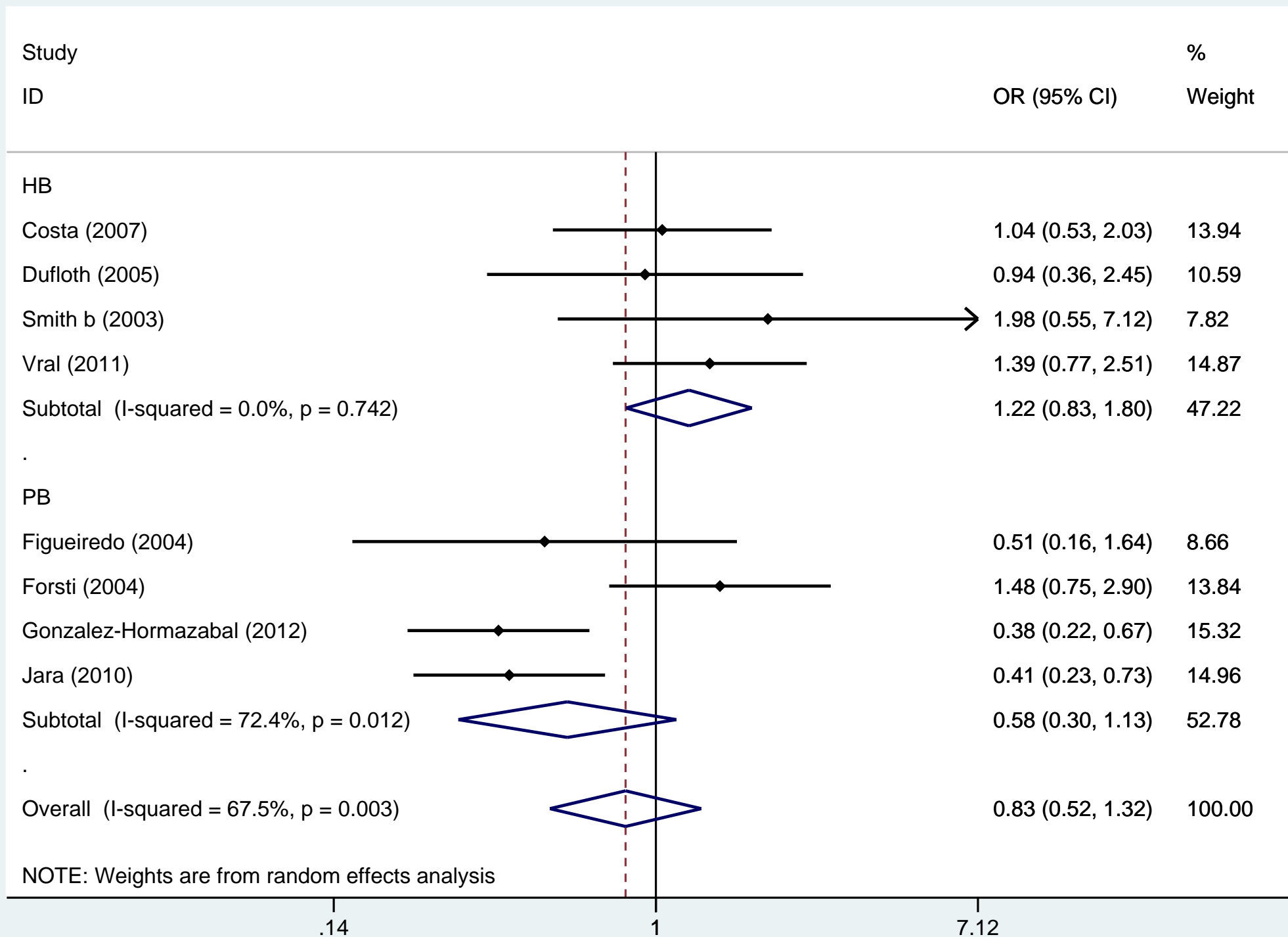

Supplement: Supplementary file 3 — Figure S2. Forest plots of XRCC3 Thr241Met polymorphism and risk of familial breast cancer in society -based subgroups. (D) Homozygote model: MM vs. TT. (E) Dominant model: TM + MM vs. TT. (F) Recessive model: MM vs. TM + TT. (ZIP 8 kb) [file 12881_2019_809_MOESM3_ESM.zip › Figure S2 CR3.pdf]

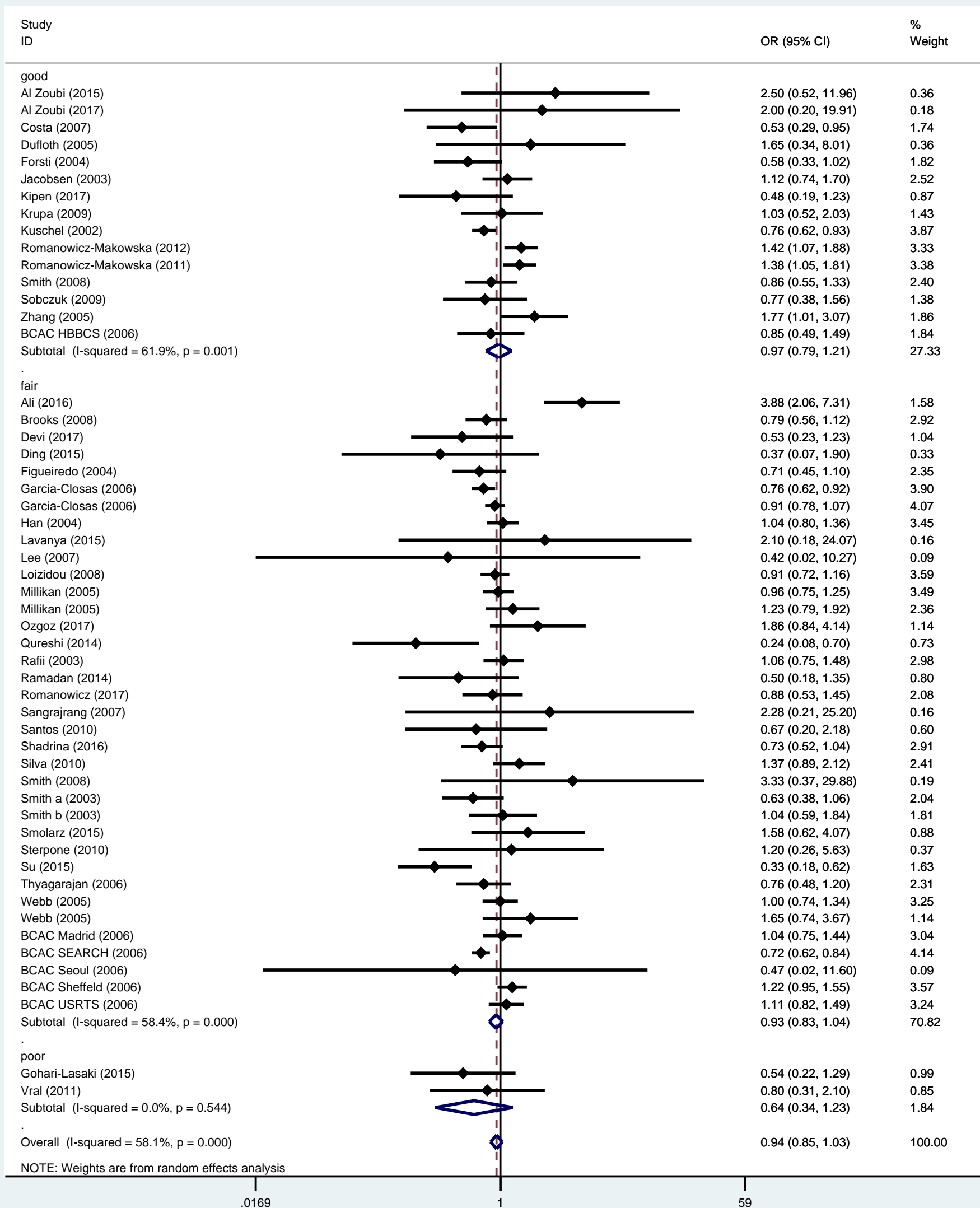

Supplement: Supplementary file 4 — Figure S3. Forest plots of XRCC3 T241 M Polymorphism and Sporadic Breast Cancer according to NOS subgroup analysis. (A) Homozygote model: MM vs. TT. (B) Dominant model: TM + MM vs. TT. (C) Recessive model: MM vs. TM + TT. (ZIP 22 kb) [file 12881_2019_809_MOESM4_ESM.zip › Figure S3 AR3.pdf]

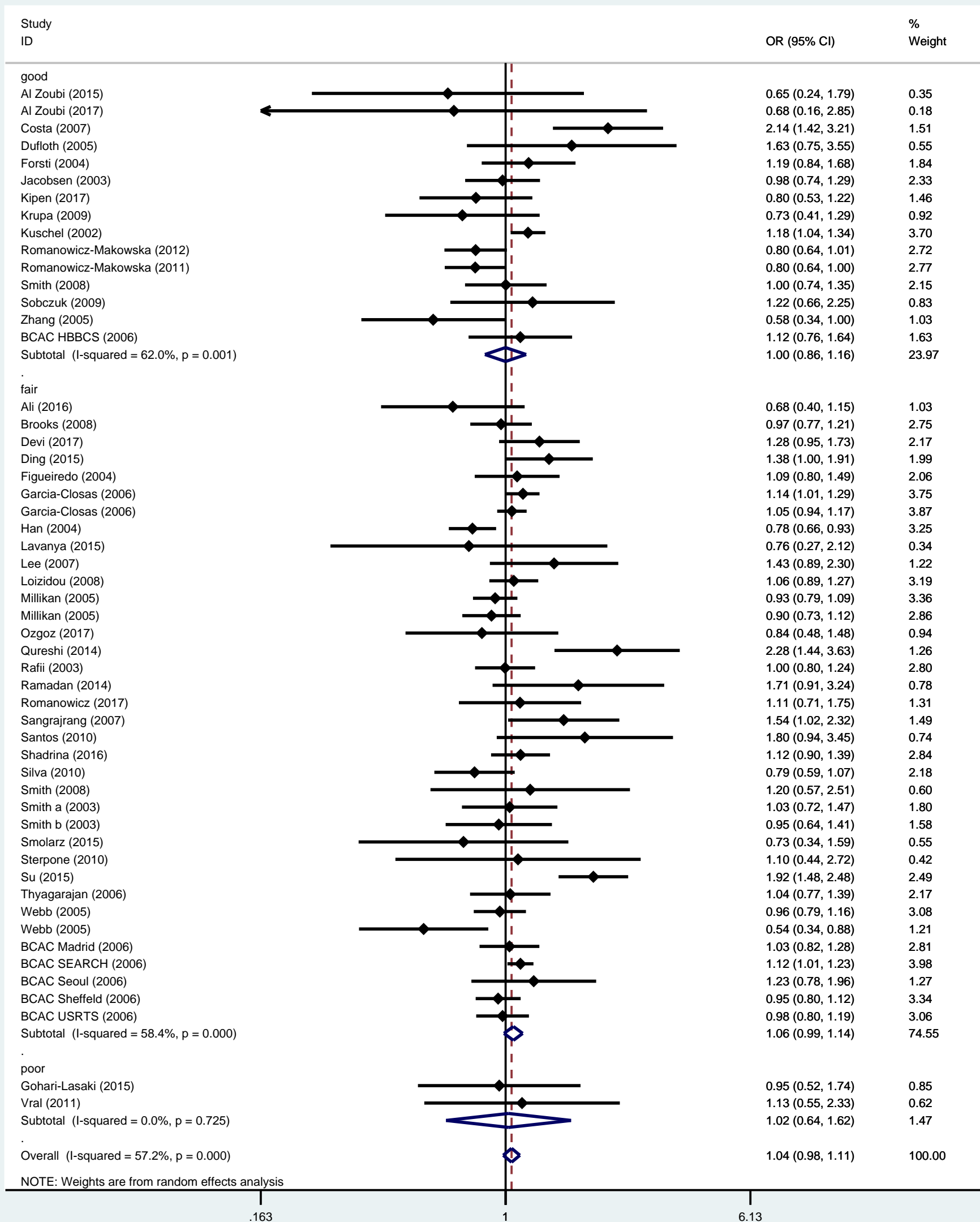

Supplement: Supplementary file 4 — Figure S3. Forest plots of XRCC3 T241 M Polymorphism and Sporadic Breast Cancer according to NOS subgroup analysis. (A) Homozygote model: MM vs. TT. (B) Dominant model: TM + MM vs. TT. (C) Recessive model: MM vs. TM + TT. (ZIP 22 kb) [file 12881_2019_809_MOESM4_ESM.zip › Figure S3 BR3.pdf]

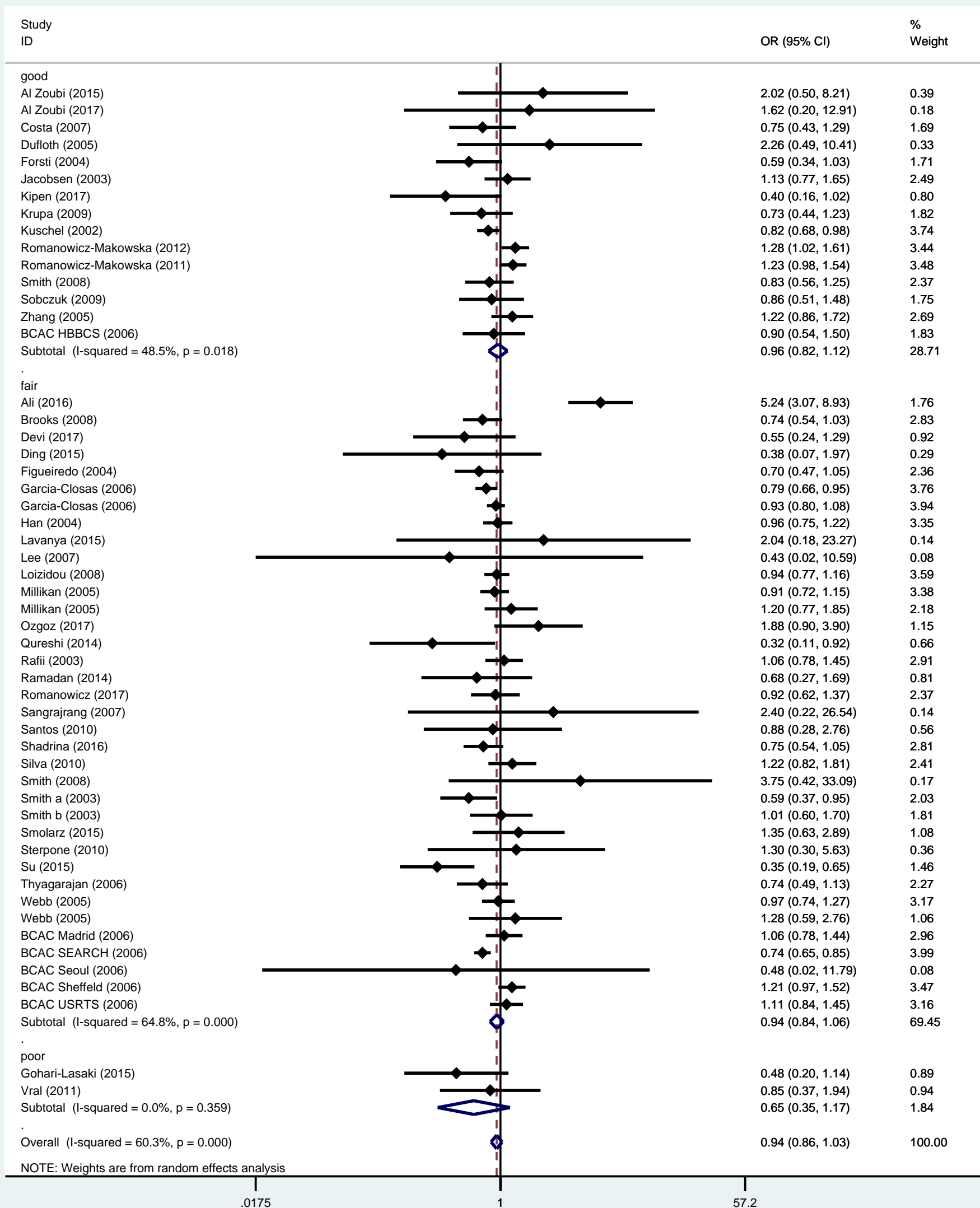

Supplement: Supplementary file 4 — Figure S3. Forest plots of XRCC3 T241 M Polymorphism and Sporadic Breast Cancer according to NOS subgroup analysis. (A) Homozygote model: MM vs. TT. (B) Dominant model: TM + MM vs. TT. (C) Recessive model: MM vs. TM + TT. (ZIP 22 kb) [file 12881_2019_809_MOESM4_ESM.zip › Figure S3 CR3.pdf]

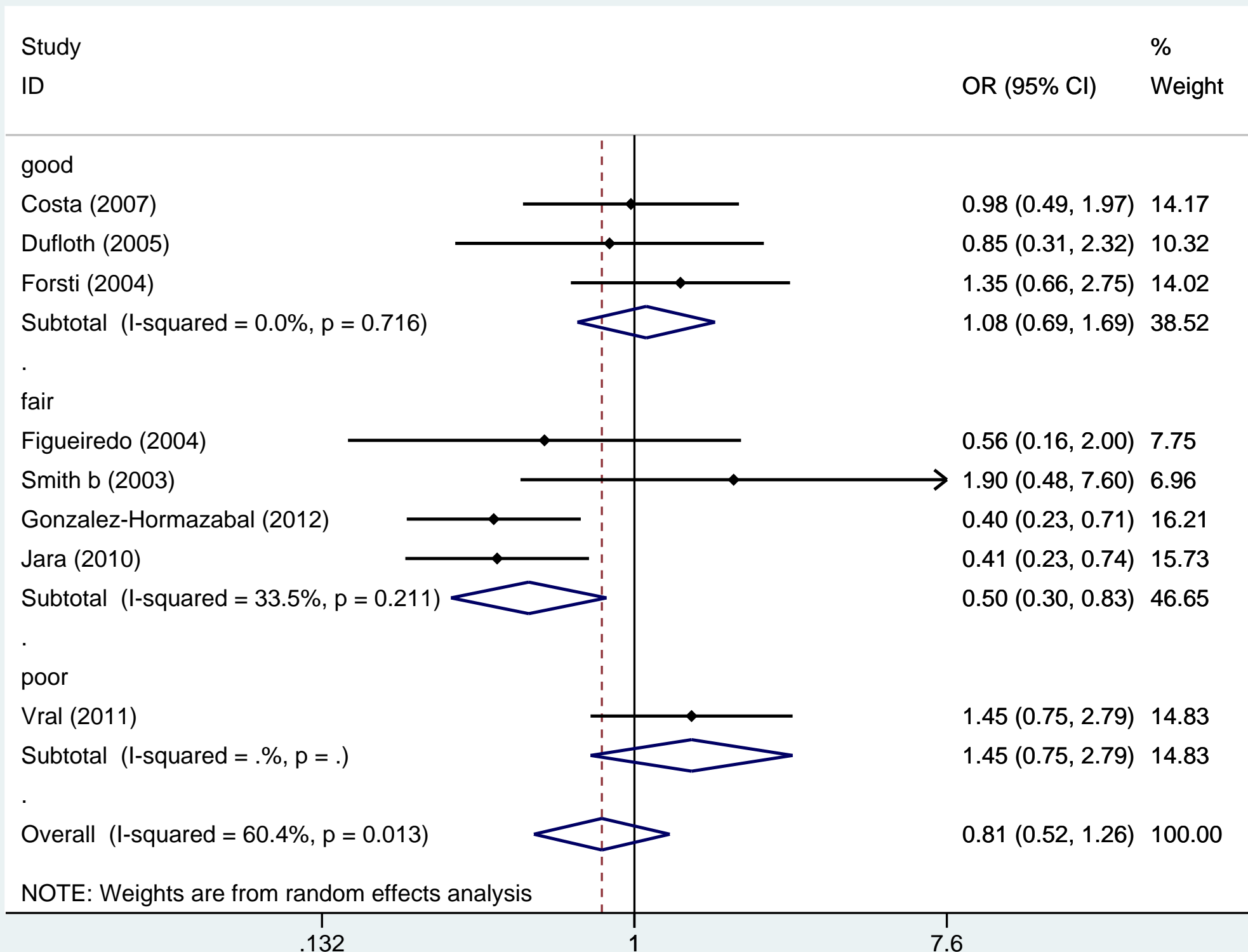

Supplement: Supplementary file 5 — Figure S4. Forest plots of XRCC3 T241 M Polymorphism and Familial Breast Cancer according to NOS subgroup analysis. (A) Homozygote model: MM vs. TT. (B) Dominant model: TM + MM vs. TT. (C) Recessive model: MM vs. TM + TT. (ZIP 9 kb) [file 12881_2019_809_MOESM5_ESM.zip › Figure S4 AR3.pdf]

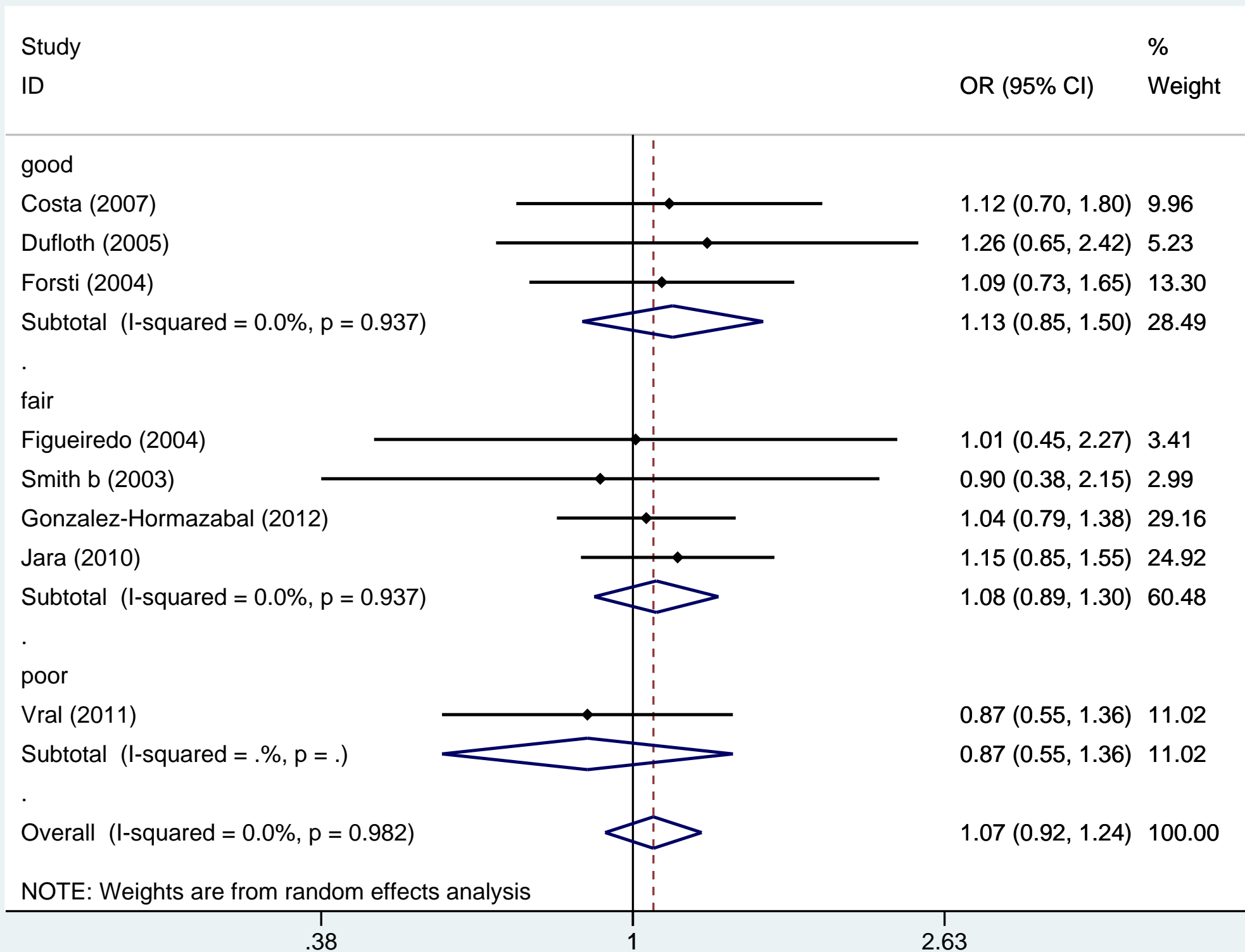

Supplement: Supplementary file 5 — Figure S4. Forest plots of XRCC3 T241 M Polymorphism and Familial Breast Cancer according to NOS subgroup analysis. (A) Homozygote model: MM vs. TT. (B) Dominant model: TM + MM vs. TT. (C) Recessive model: MM vs. TM + TT. (ZIP 9 kb) [file 12881_2019_809_MOESM5_ESM.zip › Figure S4 BR3.pdf]

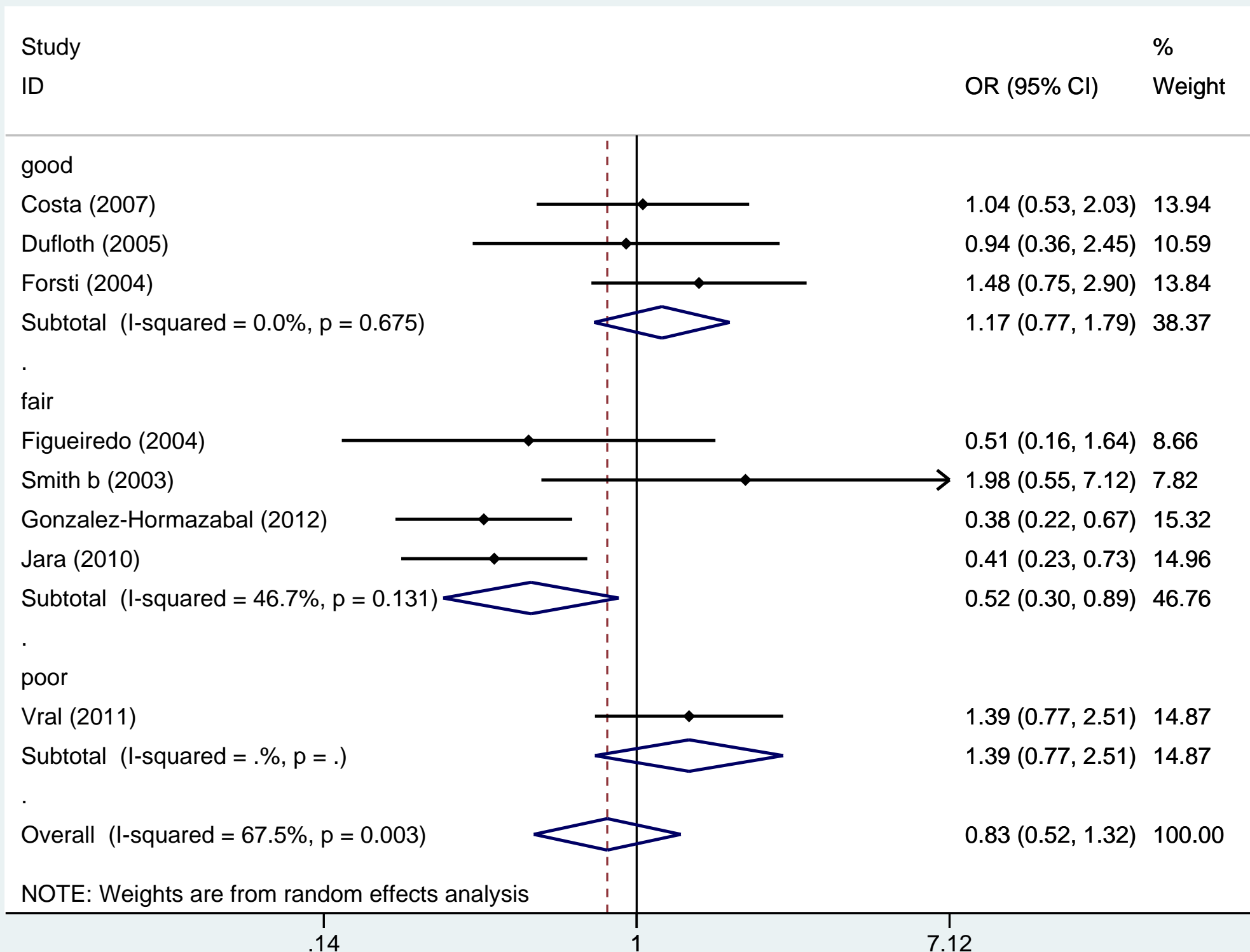

Supplement: Supplementary file 5 — Figure S4. Forest plots of XRCC3 T241 M Polymorphism and Familial Breast Cancer according to NOS subgroup analysis. (A) Homozygote model: MM vs. TT. (B) Dominant model: TM + MM vs. TT. (C) Recessive model: MM vs. TM + TT. (ZIP 9 kb) [file 12881_2019_809_MOESM5_ESM.zip › Figure S4 CR3.pdf]

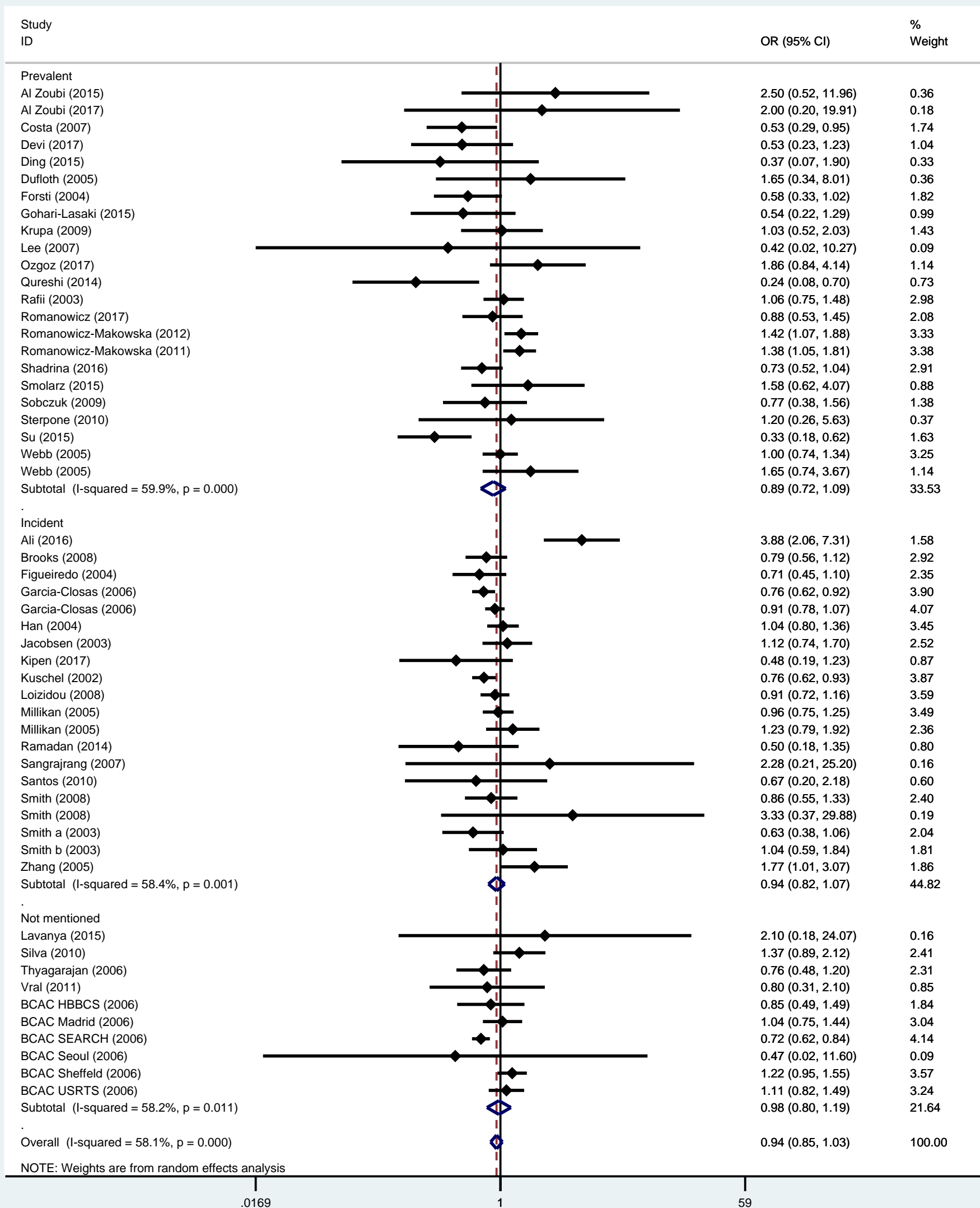

Supplement: Supplementary file 6 — Figure S5. Forest plots of XRCC3 T241 M Polymorphism and Sporadic Breast Cancer according to case enrollment subgroup analysis. (A) Homozygote model: MM vs. TT. (B) Dominant model: TM + MM vs. TT. (C) Recessive model: MM vs. TM + TT. (ZIP 22 kb) [file 12881_2019_809_MOESM6_ESM.zip › Figure S5 AR3.pdf]

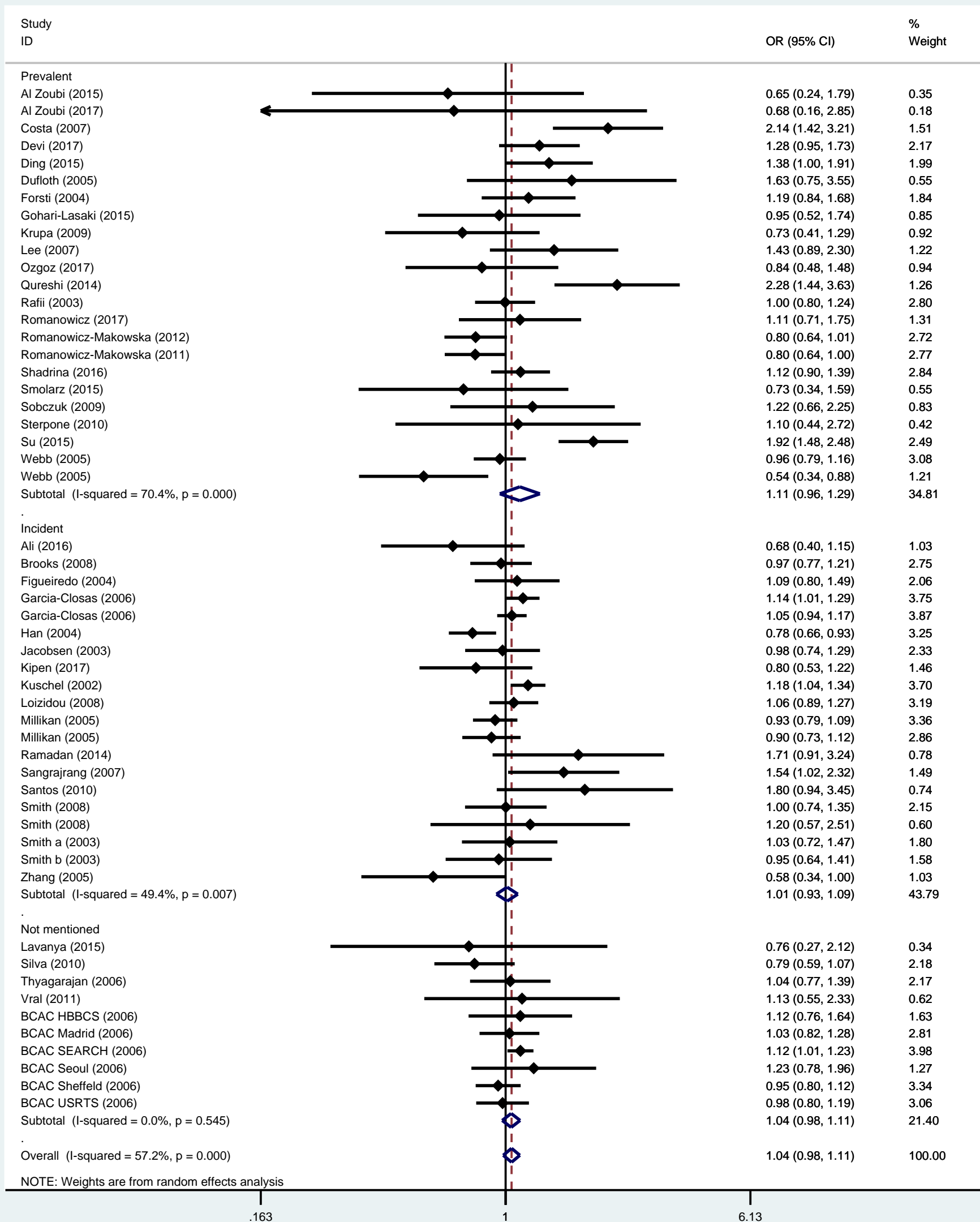

Supplement: Supplementary file 6 — Figure S5. Forest plots of XRCC3 T241 M Polymorphism and Sporadic Breast Cancer according to case enrollment subgroup analysis. (A) Homozygote model: MM vs. TT. (B) Dominant model: TM + MM vs. TT. (C) Recessive model: MM vs. TM + TT. (ZIP 22 kb) [file 12881_2019_809_MOESM6_ESM.zip › Figure S5 BR3.pdf]

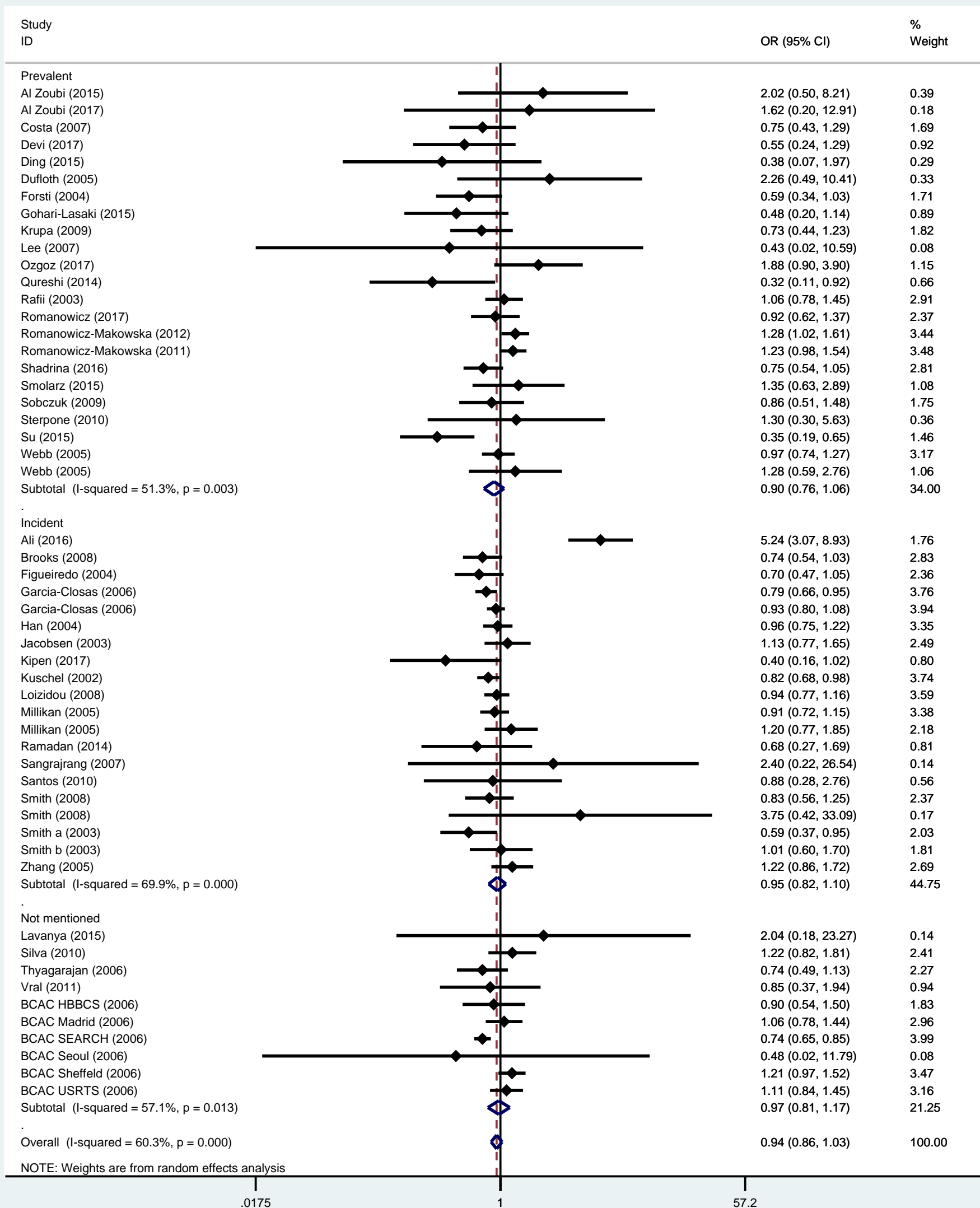

Supplement: Supplementary file 6 — Figure S5. Forest plots of XRCC3 T241 M Polymorphism and Sporadic Breast Cancer according to case enrollment subgroup analysis. (A) Homozygote model: MM vs. TT. (B) Dominant model: TM + MM vs. TT. (C) Recessive model: MM vs. TM + TT. (ZIP 22 kb) [file 12881_2019_809_MOESM6_ESM.zip › Figure S5 CR3.pdf]

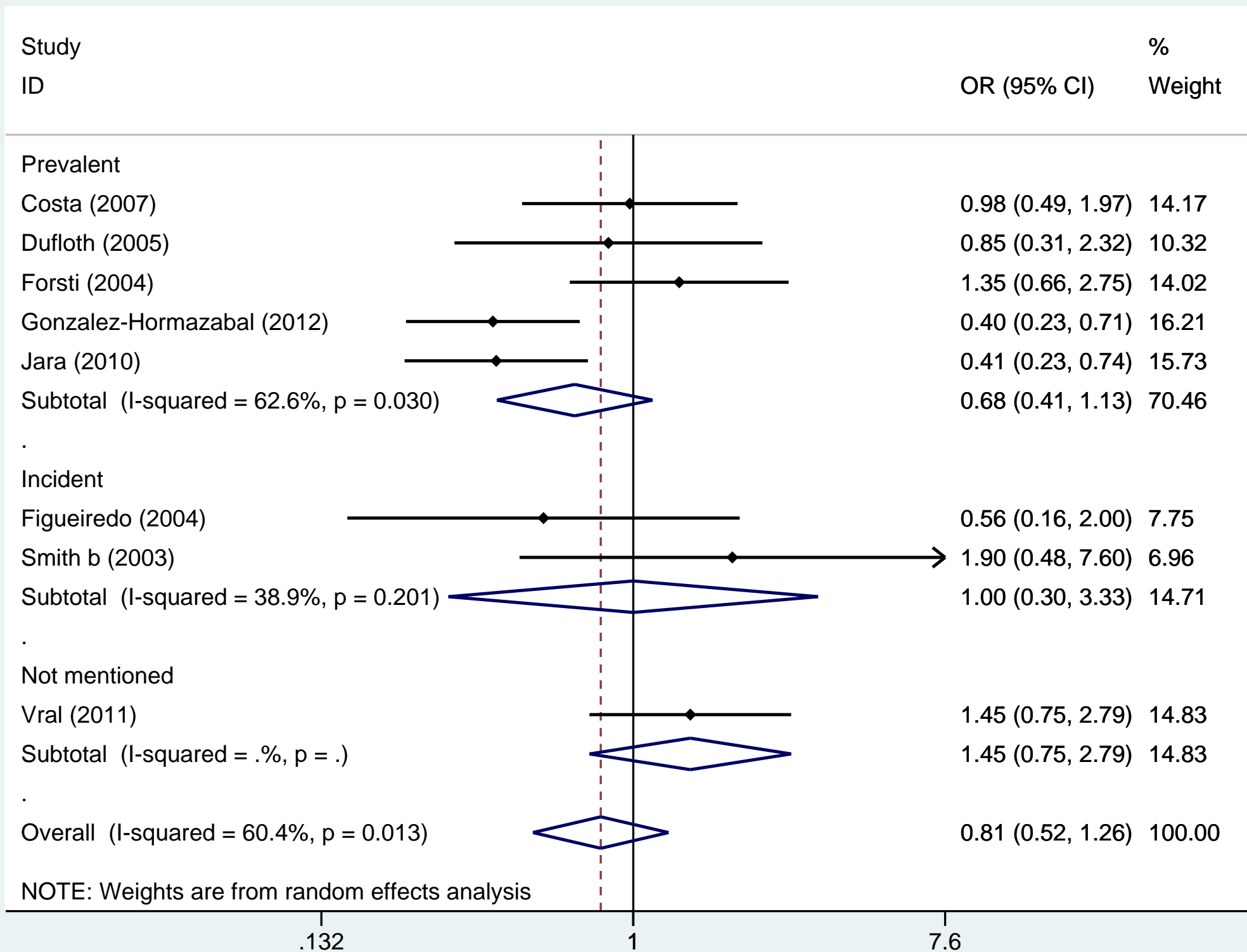

Supplement: Supplementary file 7 — Figure S6. Forest plots of XRCC3 T241 M Polymorphism and Familial Breast Cancer according to case enrollment subgroup analysis. (A) Homozygote model: MM vs. TT. (B) Dominant model: TM + MM vs. TT. (C) Recessive model: MM vs. TM + TT. (ZIP 9 kb) [file 12881_2019_809_MOESM7_ESM.zip › Figure S6 AR3.pdf]

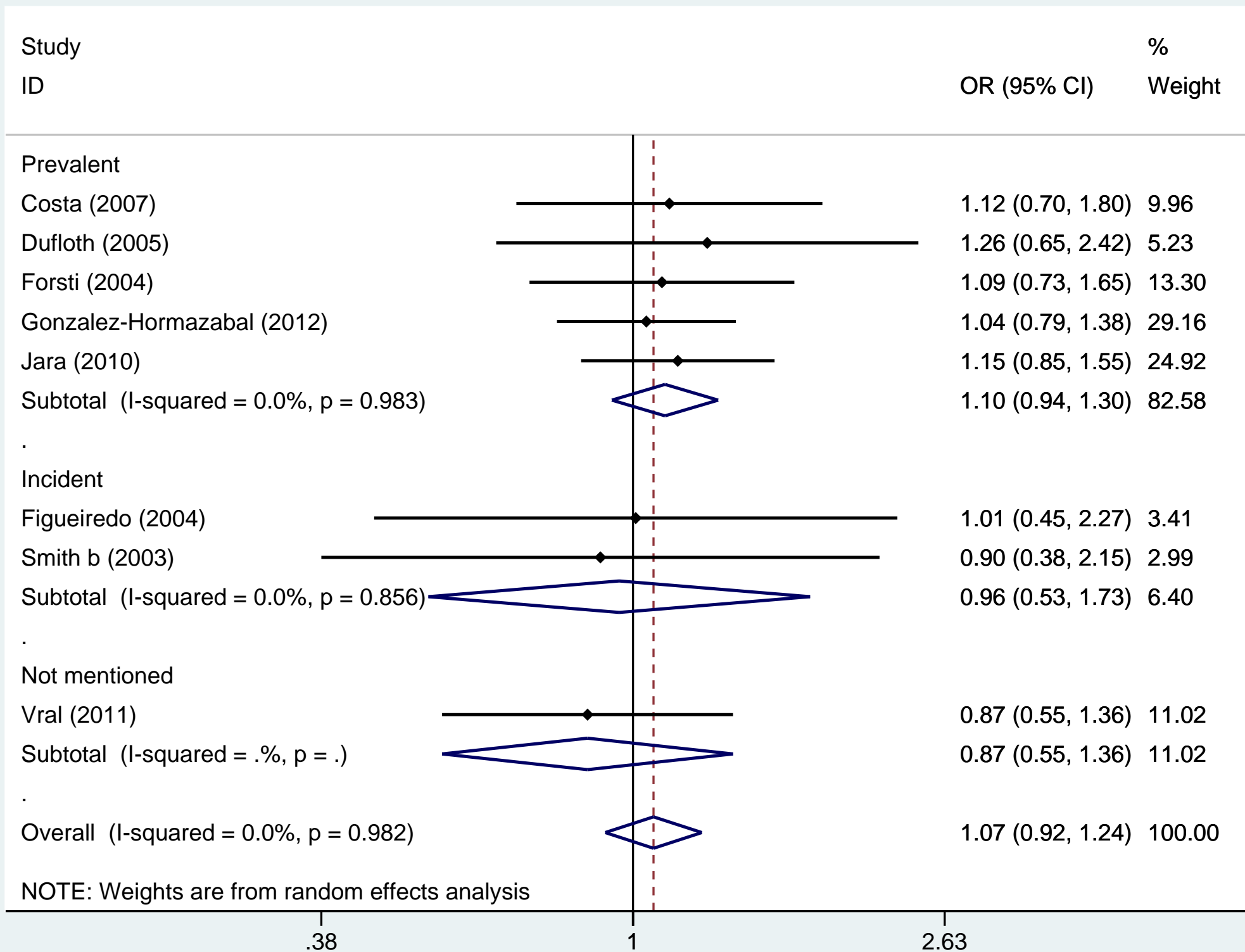

Supplement: Supplementary file 7 — Figure S6. Forest plots of XRCC3 T241 M Polymorphism and Familial Breast Cancer according to case enrollment subgroup analysis. (A) Homozygote model: MM vs. TT. (B) Dominant model: TM + MM vs. TT. (C) Recessive model: MM vs. TM + TT. (ZIP 9 kb) [file 12881_2019_809_MOESM7_ESM.zip › Figure S6 BR3.pdf]

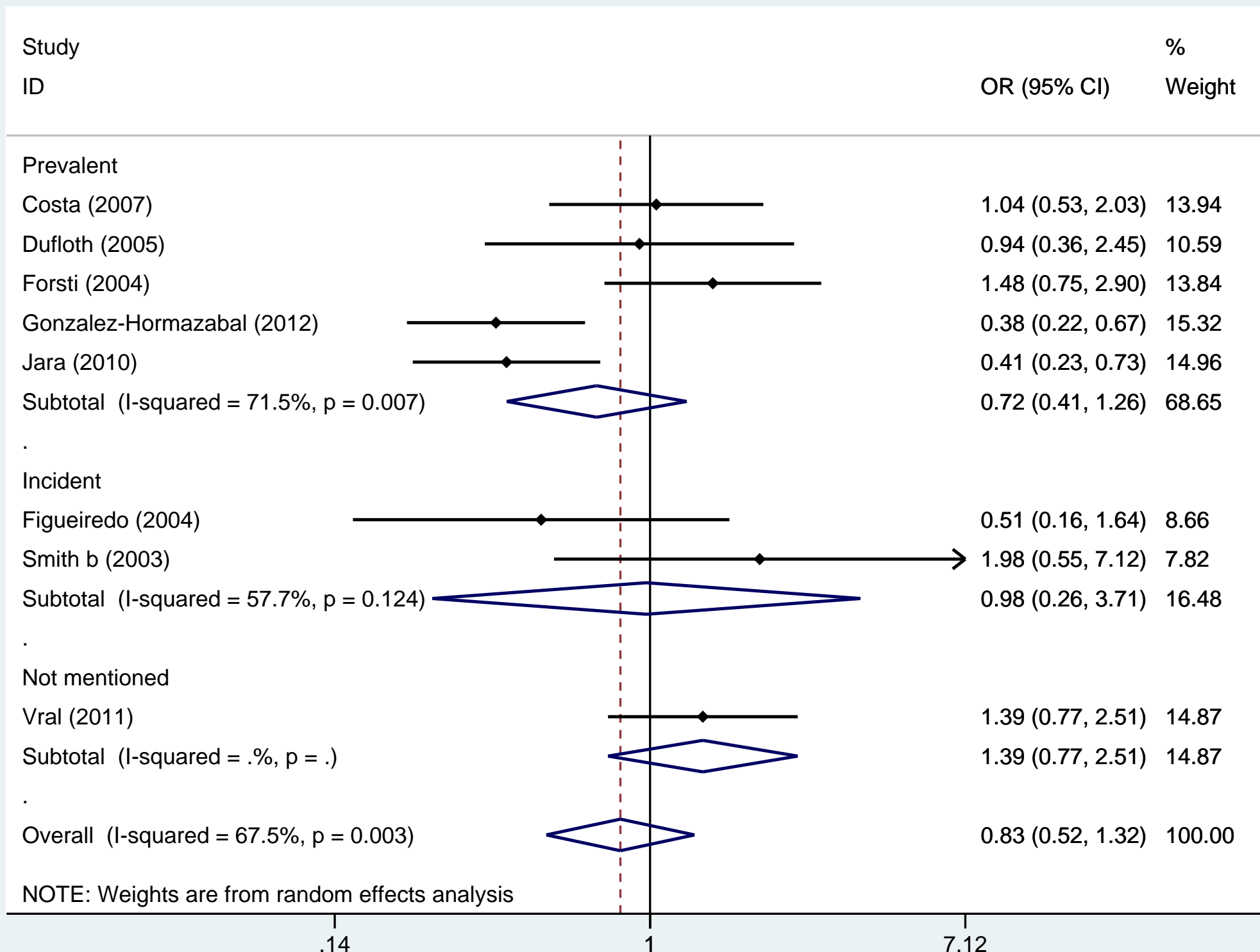

Supplement: Supplementary file 7 — Figure S6. Forest plots of XRCC3 T241 M Polymorphism and Familial Breast Cancer according to case enrollment subgroup analysis. (A) Homozygote model: MM vs. TT. (B) Dominant model: TM + MM vs. TT. (C) Recessive model: MM vs. TM + TT. (ZIP 9 kb) [file 12881_2019_809_MOESM7_ESM.zip › Figure S6 CR3.pdf]
